# Effect of naturally-occurring mutations on the stability and function of cancer-associated NQO1: Comparison of experiments and computation

**DOI:** 10.3389/fmolb.2022.1063620

**Published:** 2022-11-24

**Authors:** Juan Luis Pacheco-Garcia, Matteo Cagiada, Kelly Tienne-Matos, Eduardo Salido, Kresten Lindorff-Larsen, Angel L. Pey

**Affiliations:** ^1^ Departamento de Química-Física, Universidad de Granada, Granada, Spain; ^2^ Department of Biology, Linderstrøm-Lang Centre for Protein Science, University of Copenhagen, Copenhagen, Denmark; ^3^ Center for Rare Diseases (CIBERER), Hospital Universitario de Canarias, Universidad de la Laguna, La Laguna, Tenerife Tenerife, Spain; ^4^ Departamento de Química Física, Unidad de Excelencia en Química Aplicada a Biomedicina y Medioambiente e Instituto de Biotecnología, Universidad de Granada, Granada, Spain

**Keywords:** protein function, protein stability, genotype-phenotype correlations, computational prediction, sequence conservation

## Abstract

Recent advances in DNA sequencing technologies are revealing a large individual variability of the human genome. Our capacity to establish genotype-phenotype correlations in such large-scale is, however, limited. This task is particularly challenging due to the multifunctional nature of many proteins. Here we describe an extensive analysis of the stability and function of naturally-occurring variants (found in the COSMIC and gnomAD databases) of the cancer-associated human NAD(P)H:quinone oxidoreductase 1 (NQO1). First, we performed *in silico* saturation mutagenesis studies (>5,000 substitutions) aimed to identify regions in NQO1 important for stability and function. We then experimentally characterized twenty-two naturally-occurring variants in terms of protein levels during bacterial expression, solubility, thermal stability, and coenzyme binding. These studies showed a good overall correlation between experimental analysis and computational predictions; also the magnitude of the effects of the substitutions are similarly distributed in variants from the COSMIC and gnomAD databases. Outliers in these experimental-computational genotype-phenotype correlations remain, and we discuss these on the grounds and limitations of our approaches. Our work represents a further step to characterize the mutational landscape of NQO1 in the human genome and may help to improve high-throughput *in silico* tools for genotype-phenotype correlations in this multifunctional protein associated with disease.

## 1 Introduction

Advances in technologies for whole-genome or exome sequencing and high-throughput functional assays have increased our knowledge on the consequences of the genetic variability in humans and the relationship to disease (McInnes et al., 2021; Arnedo-Pac et al., 2022; Høie et al., 2022; Katsonis et al., 2022). However, our capacity to predict the pathogenicity of single amino acid variants is still limited, with some approaches providing good overall results but failing to predict correlation for some individual mutations or phenotypes (Katsonis et al., 2022).

Current approaches for correlating genotype and phenotype can broadly be classified into two classes. First, experimental approaches are based on the characterization of one or several functional features (for example enzymatic function and regulation, protein-protein interactions, transport to different intracellular or extracellular locations, protein turnover, ligand binding) (Xu et al., 2017; Abildgaard et al., 2019; Pacheco-Garcia et al., 2021; Høie et al., 2022). In the case of high-throughput experimental approaches typically only one or two aspects of protein function are analysed (for example protein abundance or ability to rescue a growth phenotype) (Cagiada et al., 2021). Second, the use of structure- or sequence-based methods to predict pathogenicity are becoming increasingly robust (Abildgaard et al., 2019; Arnedo-Pac et al., 2022; Høie et al., 2022). Although experiments may be implemented in a high-throughput fashion, it has until now been limited to a relatively small set of proteins and assays (Arnedo-Pac et al., 2022; Høie et al., 2022). Thus, while computational approaches also have limitations, they may be appealing due to their potential application on a proteomic scale (Arnedo-Pac et al., 2022; Høie et al., 2022).

In this work, we apply both types of approaches to increase our understanding of the correlation between genotype and phenotype for missense variants of the human NAD(P)H:quinone oxidoreductase 1 (NQO1) protein. NQO1 is associated with several diseases including cancer, Alzheimer´s and Parkinson’s disease (Beaver et al., 2019; Luo et al., 2019). NQO1 is a multifunctional protein, displaying both enzymatic and non-enzymatic functions. As an enzyme, it catalyzes the FAD-dependent (two-electron) reduction of a large set of quinone substrates, with functions including redox maintenance of vitamins, detoxification of xenobiotics and activation of cancer pro-drugs (Ross and Siegel, 2018; Beaver et al., 2019; Anoz-Carbonell et al., 2020; Salido et al., 2022). Among non-enzymatic functions, NQO1 may interact with proteins and RNA, controlling their stability and function (Beaver et al., 2019; Asher et al., 2005; Ben-Nissan and Sharon, 2014; di Francesco et al., 2016; Oh et al., 2016). Many of these functions are associated with the catalytic competence and FAD binding, such as protein-protein interactions, intracellular stability and association with microtubules (Asher et al., 2005; Martínez-Limón et al., 2016; Martínez-Limón et al., 2020; Siegel et al., 2021). The native form of NQO1 is dimeric, containing two different domains: an N-terminal domain (NTD, residues 1–225), that tightly binds one FAD molecule/domain required for catalysis and contains most of the monomer-monomer interface (MMI), whereas the C-terminal domain (CTD, residues 225–274) complete the active site and the MMI (Li et al., 1995; Faig et al., 2000; Lienhart et al., 2014; Medina-Carmona et al., 2017a; Pacheco-Garcia et al., 2021).

We have recently shown that ligand binding and variant effects on stability propagate to long distances in the native state, affecting different functional features in a counterintuitive fashion (Medina-Carmona et al., 2016; Pey, 2018; Medina-Carmona et al., 2019a; Vankova et al., 2019; Pacheco-García et al., 2020; Pacheco-Garcia et al., 2021; Pacheco-Garcia et al., 2022a). Therefore, NQO1 represents a challenging and biomedically relevant system to compare the performance of computational and experimental methods to explain and to predict genotype-phenotype in a large-scale for a multi-functional protein. Here, we use computational tools to probe 5,187 variants of NQO1 that includes a set of clinically relevant missense variants which we then experimentally characterized. In this set, thirteen variants come from large-scale human sequencing data (gnomAD) and nine from the catalogue of somatic mutations in human cancer lines (COSMIC) (Table 1). As of ninth of January 2022, none of these variants were found in both databases. Whether variants found in COSMIC or gnomAD databases are associated with disease (e.g. predisposition to cancer development) is unknown. The set of variants we studied experimentally comprises very different amino acid side chain characteristics and display different levels of solvent exposure (Table 1).

**TABLE 1:** Set of NQO1 variants experimentally characterized in this work.

Mutation	Database	% ASA^a^	Variant class	Residue class
G3S	gnomAD	6.0 ± 4.7	WT-like	WT-like
G3D	COSMIC	6.0 ± 4.7	WT-like	WT-like
L7P	COSMIC	0.3 ± 0.2	Total-loss	Total-loss
L7R	gnomAD	0.3 ± 0.2	Total-loss	Total-loss
V9I	gnomAD	0.0 ± 0.0	WT-like	Total-loss
T16M	gnomAD	43 ± 14	Stable but inactive	WT-like
Y20N	gnomAD	21 ± 5	WT-like	WT-like
A29T	COSMIC	2.2 ± 0.5	WT-like	Unstable but active
K32N	gnomAD	79 ± 11	WT-like	WT-like
G34V	gnomAD	54 ± 11	Total-loss	Stable but Inactive
E36K	gnomAD	64 ± 3	WT-like	WT-like
S40L	gnomAD	0.0 ± 0.0	WT-like	Total-loss
D41G	gnomAD	14 ± 2	Total-loss	Stable but inactive
D41Y	COSMIC	14 ± 2	Stable but inactive	Stable but inactive
M45L	COSMIC	28 ± 5	WT-like	WT-like
M45I	COSMIC	28 ± 5	WT-like	WT-like
I51V	gnomAD	14 ± 1	WT-like	Stable but inactive
W106R	gnomAD	10 ± 1	Total-loss	Total-loss
W106C	COSMIC	10 ± 1	Total-loss	Total-loss
F107C	gnomAD	7.6 ± 0.5	Unstable but active	Unstable but active
M155I	COSMIC	13 ± 3	WT-like	WT-like
H162N	COSMIC	6.6 ± 0.5	WT-like	WT-like

The table indicates whether the variants are found in the COSMIC/gnomAD databases as well as the solvent exposure (as % ASA) determined using a crystallographic model of WT NQO1 [PDB code 2F1O (Asher et al., 2006)], the software Getarea and the computational classification at variant and residue level using a combination of predictions of thermodynamic stability change upon mutation and evolutionary conservation.

^a^ Using GetArea (http://curie.utmb.edu/getarea.html) and the structure with PDB code 2F1O (Asher et al., 2006). Data are the average ±s.d. from eight monomers.

## 2 Materials and methods

### 2.1 Experimental methods

#### 2.1.1 Protein expression and purification

Mutations were introduced by site-directed mutagenesis in the wild-type (WT) NQO1 cDNA cloned into the pET-15b vector (pET-15b-NQO1) by GenScript (Leiden, Netherlands). Mutated codons were optimized for expression in *E. coli* and mutagenesis was confirmed by sequencing the entire cDNA. The plasmids were transformed in *E. coli* BL21 (DE3) cells (Agilent Technologies, Santa Clara, CA, United States) for protein expression.

To determine the amount of soluble NQO1 at 37°C, 5 ml of LB medium containing 0.1 mg mL^−1^ ampicillin (purchased from Canvax Biotech, Córdoba, Spain) was inoculated with transformed cells and grown for 16 h at 37°C. 0.5 ml of these cultures were diluted into 10 ml of LB containing 0.1 mg mL^−1^ ampicillin (LBA) and grown at 37°C for 3 h. After that, cultures were induced with 0.5 mM of isopropyl β-D-1-thiogalactopyranoside (IPTG, Canvax Biotech) at 37 °C for 4 h. Cells were harvested by centrifugation at 2,900 *g* in a bench centrifuge at 4°C and frozen at −80°C for 16 h. Pellets were resuspended in binding buffer (20 mM Na-phosphate, 300 mM NaCl, 50 mM imidazole, pH 7.4) with 1 mM phenylmethylsulfonyl fluoride (PMSF, Sigma-Aldrich, Madrid, Spain) and sonicated in an ice bath. These *total extracts* were centrifugated (24,000 *g*, 30 min, 4°C in a bench centrifuge) to obtain the *soluble extracts*. The amount of NQO1 in total and soluble extracts was determined by Western-blotting providing the S/T (soluble/total) ratio for each variant. Samples were denatured using Laemmli’s buffer, resolved in polyacrylamide gel electrophoresis in the presence of sodium dodecylsulphate (SDS-PAGE, 12% acrylamide) gels and transferred to PVDF membranes (GE Healthcare, Chicago, IL, United States) using standard procedures. Immunoblotting was carried out using primary monoclonal antibody against NQO1 (sc-393736, Santa Cruz Biotechnology, Dallas, TX, United States) at 1:500 dilution and, as secondary antibody, an anti-mouse IgGκ BP-HRP (sc-516102, Santa Cruz Biotechnology) at 1:2000 dilution was used. Samples were visualized using luminol-based enhanced chemiluminescence (from BioRad Laboratories, Hercules, CA, United States), scanned and analysed using Image Lab (from BioRad Laboratories).

For large-scale purifications, a preculture (100 ml) was prepared from a single clone for each variant and grown for 16 h at 37°C in LBA and diluted into 2.4–4.8 L of LBA. After 3 h at 37°C, NQO1 expression was induced by the addition of 0.5 mM IPTG for 6 h at 25°C. Cells were harvested by centrifugation at 8,000 *g* and frozen overnight at −80 °C. NQO1 proteins were purified using immobilized nickel affinity chromatography columns (IMAC, GE Healthcare) as described (Anoz-Carbonell et al., 2020). Isolated dimeric fractions of NQO1 variants were exchanged to HEPES-KOH buffer 50 mM pH 7.4 using PD-10 columns (GE Healthcare). The UV–visible spectra of purified NQO1 proteins were measured in a Cary spectrophotometer (Agilent Technologies, Waldbronn, Germany) and used to quantify NQO1 concentration and the content of FAD as described in (Anoz-Carbonell et al., 2020). Apo-proteins were obtained as described in (Vankova et al., 2019). Briefly, holo-proteins were incubated with 2 M urea and 2 M KBr in HEPES-KOH 50 mM pH 7.4 in the presence of 2 mM β-mercaptoethanol and 1 mM PMSF and loaded into IMAC columns, washed with 2 M urea and 2 M KBr in HEPES-KOH 50 mM pH 7.4, 2 mM β-mercaptoethanol, then with HEPES-KOH 50 mM pH 7.4, 2 mM β-mercaptoethanol eluted with 20 mM Na-Phosphate 300 mM NaCl 500 mM imidazole pH 7.4 and finally exchanged to HEPES-KOH 50 mM pH 7.4. These apo-proteins contained less than 2% bound FAD based on UV-visible spectra. Samples were stored at −80°C upon flash freezing in liquid N_2_. Protein purity and integrity was checked by SDS-PAGE.

#### 2.1.2 *In vitro* characterization of NQO1 variants

Thermal denaturation of NQO1 proteins, as holo-proteins (2 μM in monomer +100 μM FAD) was monitored by following changes in tryptophan emission fluorescence in HEPES-KOH 50 mM at pH 7.4 as described in (Medina-Carmona et al., 2017b). *T*_m_ values were reported as mean ± s.d. of four independent measurements.

Fluorescence titrations were carried out at 25°C using 1 cm × 0.3 cm path-length cuvettes in a Cary Eclipse spectrofluorimeter (Agilent Technologies, Waldbronn, Germany). Experiments were performed in 20 mM K-phosphate, pH 7.4, essentially as described in (Pacheco-García et al., 2020). Briefly, apo-NQO1 (0.25 µM in subunit) was mixed with 0–2 μM FAD in K-phosphate 20 mM pH 7.4. Samples were incubated at 25°C in the dark for at least 10 min before measurements. Spectra were acquired in the 340–360 nm range upon excitation at 280 nm (slits 5 nm), and spectra were averaged over 10 scans registered at a scan rate of 200 nm min^−1^. FAD binding fluorescence intensities at 350 nm were fitted using a single and identical type of binding sites as described in (Pacheco-García et al., 2020). Variant effects on the FAD binding free energy (ΔΔG_FAD_) were calculated as:

$$∆∆G_{FAD}=R\cdot T\cdot \ln\frac{K_{d\left(mut\right)}}{K_{d\left(WT\right)}}$$ (1)

Where R is the ideal gas constant (1.987 cal mol·K^−1^), T is the experimental temperature (298.15 K), and *K*_d(mut)_ and *K*_d(WT)_ are the FAD binding dissociation constant of the mutant and WT protein variants, respectively. A positive value of ΔΔG_FAD_ indicates that the mutation reduces the affinity for FAD.

For proteolysis studies, NQO1 samples (10 μM in monomer) were prepared in HEPES-KOH 50 mM at pH 7.4 in the presence of 100 μM FAD (NQO1_holo_) and incubated at 25^o^C for 5 min. The proteolysis reaction was initiated upon addition of 0.02–1.2 μM thermolysin (from *Geobacillus stearothermophilus*, Sigma-Aldrich) and a final concentration of 10 mM CaCl_2_. Samples were incubated at 25°C and aliquots were collected over time and the reaction quenched by addition of EDTA pH 8 (final concentration of 20 mM) and Laemmli´s buffer (2x). Controls (time 0) were prepared likewise but without thermolysin. Samples were resolved by SDS-PAGE under reducing conditions in gels containing 12% acrylamide. Gels were stained with Coomassie blue G-250. Densitometry was carried out using ImageJ. Data were analyzed using an exponential function to provide the apparent rate constant (*k*_obs_). From the linear dependence of *k*_obs_ vs. [thermolysin], we obtained the second-order rate constant *k*_prot_. Linearity in these plot indicate that proteolysis occurs under a EX2 mechanism, thus reflecting the thermodynamic stability of the thermolysin cleavage site (Ser72-Val73) between non-cleavable and cleavable states (Park and Marqusee, 2004). These *k*_prot_ values were used to determine mutational effects on the local stability of thermolysin cleave site (ΔΔG_PROT_) using Eq. 2:

$$∆∆G_{PROT}=R\cdot T\cdot \ln\frac{k_{prot\left(mut\right)}}{k_{prot\;\left(WT\right)}}$$ (2)

Where R is the ideal gas constant (1.987 cal mol·K^−1^), T is the experimental temperature (298.15 K), and *k*_prot(mut)_ and *k*_prot(WT)_ are the second-order proteolysis rate constants of the mutant and WT protein variants, respectively. A positive value of ΔΔG_PROT_ indicates that the mutation thermodynamically destabilizes the thermolysin cleavage site.

### 2.2 Computational analyses

#### 2.2.1 Evolutionary conservation analysis

We used GEMME (Laine et al., 2019) to evaluate evolutionary distances from the WT NQO1 sequence (Uniprot ID: P15559 — isoform 1). We used HHblits (Remmert et al., 2011; Steinegger et al., 2019) to generate a multiple sequence alignment (MSA) using UniClust30 as sequence database and an E-value threshold of 10^−10^. The resulting MSA contained 1,602 sequences and was refined using two additional filters: first, all the columns that were not present in the WT NQO1 sequence were removed; second, all the sequences with more than the 50% of gaps were removed. Application of these two filters yielded 1,414 sequences in the MSA. The GEMME package was run using default parameters. For each position, a median score was evaluated using all the available substitutions.

#### 2.2.2 Thermodynamic stability predictions

Changes in thermodynamic stability (ΔΔG) were calculated using the crystal structure (Faig et al., 2000) (PDB 1D4A) and the Rosetta package (GitHub SHA1 c7009b3115c22daa9efe2805d9d1ebba08426a54) with the Cartesian ΔΔG protocol (Park et al., 2016; Frenz et al., 2020). The values obtained from Rosetta in internal Energy Unit were divided by 2.9 to bring them on to a scale corresponding to kcal·mol^−1^ (Park et al., 2016). Median scores were evaluated for each position using all the available substitutions.

We used DSSP (Kabsch and Sander, 1983) to calculate the solvent accessible surface area (SASA) when identifying interface residues in NQO1. Interface residues were defined as those residues for a difference larger than 0.2 was detected between SASA calculations based on the dimer and monomer structure.

## 3 Results

### 3.1 Saturation mutagenesis by computational methods

We first used the predictive ability of evolutionary conservation analysis combined with thermodynamic stability calculations to classify all possible variants (i.e. saturation mutagenesis) in NQO1 based on their effects on the protein function and stability (Cagiada et al., 2021). For evolutionary conservation studies, we used GEMME (Laine et al., 2019) which provides a score (ΔE) for all possible single amino acid change variants of NQO1 (Supplementary Figure S1A). ΔE represents the evolutionary distance of a variant from the WT NQO1 sequence, and ΔE has been shown to be a useful predictor of the deleterious effects on function and stability of the given substitution. We used Rosetta (Park et al., 2016) to predict variant effects on thermodynamic stability (ΔΔG) using a crystal structure of NQO1 (Faig et al., 2000) as input (Supplementary Figure S1B) and subsequently calculated the median ΔΔG for all variants at each position. We performed ΔΔG evaluations using both the monomeric and dimeric structure of NQO1 to separate effects on overall stability and effects on dimerization. Specifically, we calculated ΔΔG from the monomer (Supplementary Figures S1B,S2A) to predict the change in thermodynamic stability relative to wild type of each variant. We also performed similar ΔΔG calculations using the dimer structure as input (Supplementary Figures S1C,S2B), introducing each missense variant in both chains (i.e., treating this as a homodimer) and used the resulting values to compare with experiments. Based on these two calculations, we also evaluated the ΔΔG of dimerization as the difference between the two Rosetta runs (Supplementary Figures S2C,D) to highlight which residues are involved in stabilizing the dimer and thus also those variants that might weaken dimer formation. Then, we evaluated the difference in the SASA between the dimeric and monomeric residues of NQO1 (Supplementary Figure S2C) and we classified 33 of them as interface residues. We found that for 20 of these interface residues the median ΔΔG of dimerization was >1 kcal mol^−1^. Of these 20, 15 were stable upon mutation in the monomeric form (median ΔΔG <2 kcal mol^−1^) and a subset of seven display a median ΔΔG of dimerization >2 kcal mol^−1^.

**FIGURE 1:**
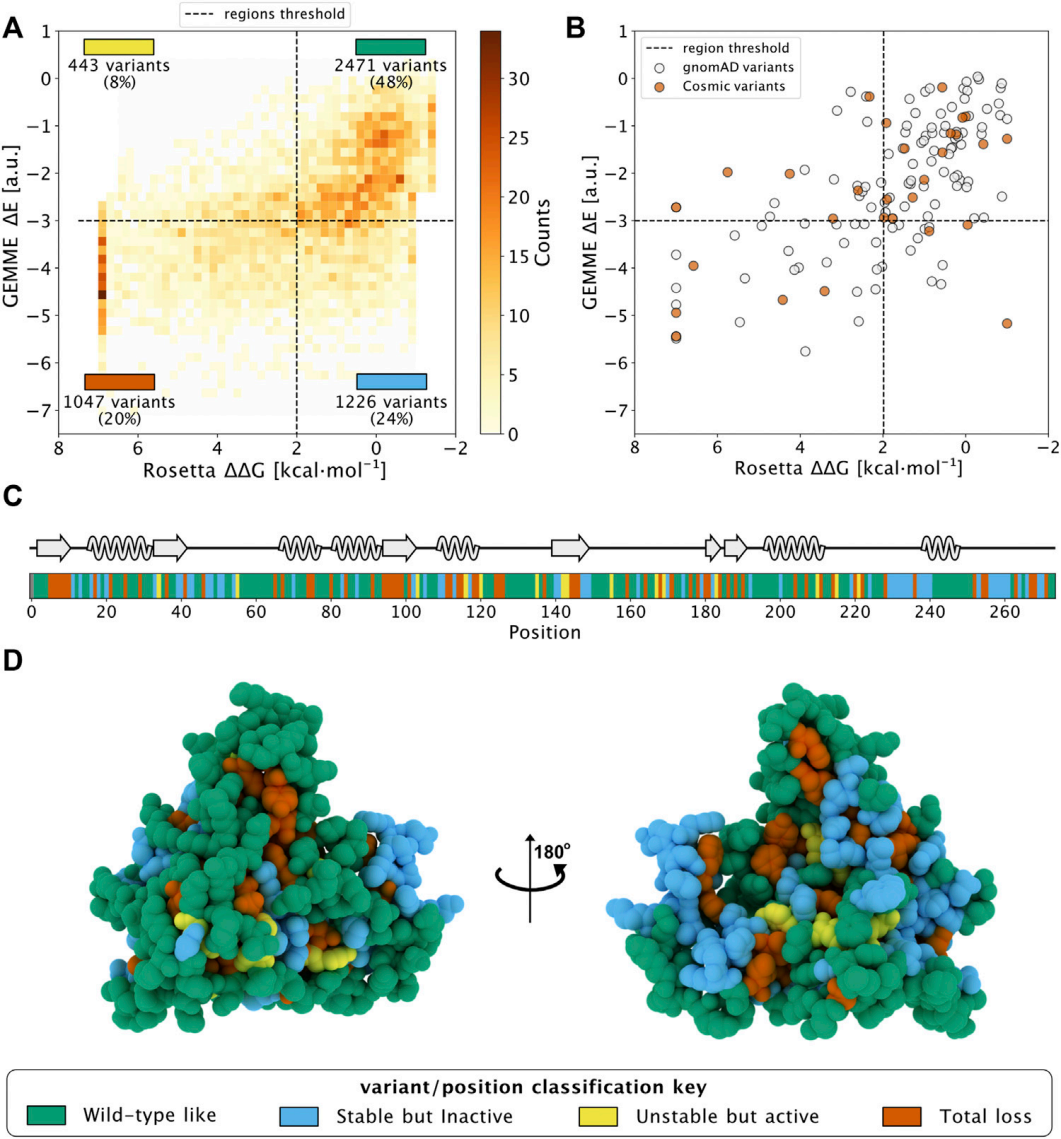
Saturation mutagenesis of NQO1 based on computational methods. **(A)** Two-dimensional histogram which combines the data from the evolutionary conservation analysis (ΔE, *y*-axis) with the thermodynamic stability (ΔΔG, *x*-axis) data from Rosetta on the NQQ1 monomer. The variants are categorized in one of the four regions, which are delimited by dashed lines. The fraction of variants, class and colour assigned to each region are indicated. The four classes of variants/positions are reported at the bottom of the figure: “WT-like” (Green), “Low stability, active” (yellow), “Stable but inactive” (blue) and “Total-loss” (red). **(B)** The scores of gnomAD (grey) and COSMIC (orange) variants in the 2D histogram. **(C)** The positional colour categories assigned using the most common colour of the position in the sequence together with the secondary structure. **(D)** The positional classification mapped to the protein crystal structure (PDB: 1D4A) (Faig et al., 2000).

Then, we combined the evolutionary conservation scores and stability calculations based on the monomeric protein for the 5,187 variants of NQO1 and plotted the results in a two-dimensional histogram (Figure 1A). We used cutoff values of 2 kcal mol^−1^ for Rosetta ΔΔG values and -3 for GEMME ΔE scores as thresholds for all the variants in order to separate them based on their effects (Luo et al., 2019). To ease analyses and interpretations, we associated each of the four defined regions with a color (Cagiada et al., 2021). ‘WT-like’ variants represent 48% of the available NQO1 variants (shown in green). 20% of NQO1 substitutions show high ΔΔG and high evolutionary distances and are classified as “Total-loss.” These variants have substitutions that are unlikely in the evolutionary analysis (ΔE < −3) and lead to decreased stability (ΔΔG >2 kcal mol^−1^); they thus likely compromise protein function *via* loss of protein stability (colored in red). Variants with high negative ΔE and low ΔΔG belong to the “Stable but inactive” class (colored in blue). This class contains 24% of the variants and represent those for which the evolutionary and stability analysis suggests that the protein function has been compromised, but not for stability reasons. Lastly, the remaining 8% of the variants show low stability and low evolutionary distance, and were classified as “Unstable but active” (colored in yellow). Having predicted the effects of all possible missense variants, we performed a similar classification of amino acid positions, assigning the most common variant class to each position (Figures 1C,D) and found 48% of the total positions classified as “WT-like,” 25% as “-Total-loss,” 22% as “Stable but inactive” and 5% as “Unstable but active.”

In addition, we used the data from the dimer analysis to evaluate the number of residues involved in the stabilization of the dimer form. We found that 14 residues at the interface changed their classification to “Total-loss” if ΔΔG was evaluated using the dimer structure. Of these 14, 9 were classified as “Stable but inactive,” while 5 were classified as WT-like using monomeric ΔΔG data. Thus, many residues at the interface appear to be conserved during evolution to preserve the stability of the dimeric form of NQO1.

Having analyzed all possible missense variants, we next looked at the results for a subset of variants that have been found in the human population. Specifically, we looked at variants that are found in the COSMIC (COSMIC v.92; https://cancer.sanger.ac.uk/cosmic) or gnomAD (gnomAD v.2.1.1; https://gnomad.broadinstitute.org/) databases, and did not find clear differences between these two sets (Figure 1B). In particular, we found variants in both sets that would be predicted as functional and others for which stability and/or conservation analyses predict loss-of-function (LoF). This result is in line with the notion that both databases may contain both potentially pathogenic as well as benign variants.

### 3.2 Selection of NQO1 variants to be experimentally characterized

**FIGURE 2:**
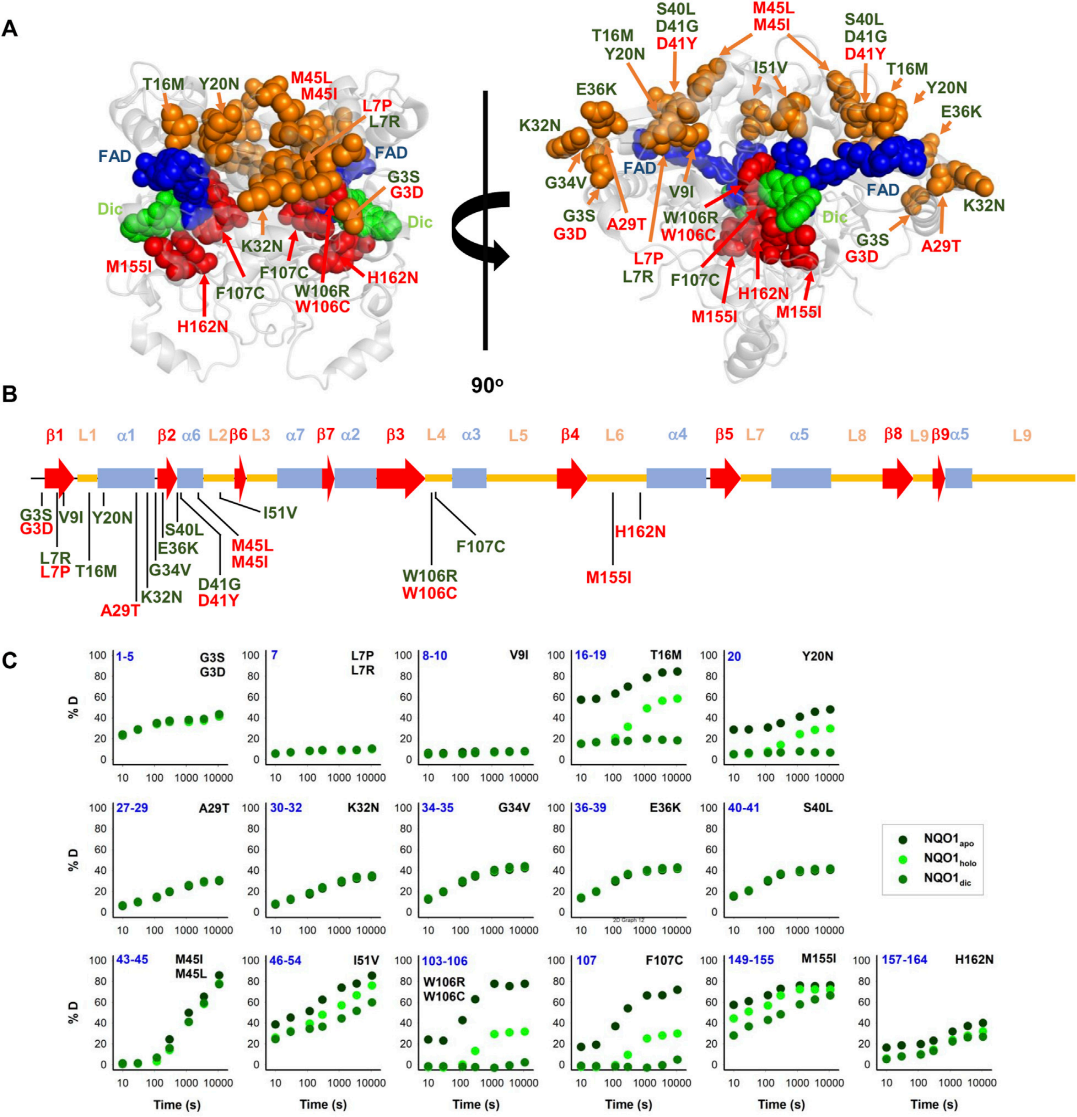
Structural features and local stability of the substituted residues and the variants characterized experimentally in this work. **(A)** The residues mutated were mapped onto the structure of NQO1 (PDB code 2F1O) (Asher et al., 2006). Residues are depicted as spheres, and the colour code indicates substitutions located in the 1–51 region (orange) or the active site (red). Variants were labelled in red (from COSMIC) or in green (from gnomAD). FAD and Dic are shown as dot representations in cyan and blue, respectively. **(B)** Location of substitutions in the sequence of NQO1 regarding secondary structure elements (from (Faig et al., 2000)). Substitutions were labelled in red (from COSMIC) or in green (from gnomAD). **(C)** HDX of segments containing the 22 variants experimentally investigated in this work. The segments are labelled in blue. The colour code corresponds to HDX for NQO1_apo_, NQO1_holo_ and NQO1_dic_ states. HDX data are from (Vankova et al., 2019).

After studying the NQO1 variants computationally, we next examined a set of the variants using a series of different experiments. In this study, we have thus extended our previous work on 8 naturally-occurring variants in NQO1 (Pacheco-García et al., 2020) to a set of 22 variants (Table 1; Figure 2). Overall, this set included thirteen variants found in the gnomAD database and nine variants found in the COSMIC database. Seventeen of these variants clustered in the N-terminal part of the protein (residues 1–51), whereas five were located in the segment comprising residues 106–162 (in close proximity to the active site). Nine variants affected residues buried inside the protein structure (with less than 10% of SASA), whereas the rest are at positions that are partially or highly solvent-exposed (Table 1). The chemical nature of the changes introduced by the substitutions is also quite diverse, and the substitutions are located in different elements of secondary structure (Figure 2B). Based on our computational analysis the 22 variants represent well the heterogeneous scale of effects on NQO1 function and stability. Indeed, of the 22 variants selected 14 are classified by the computational models as “WT-like,” 4 as “Total-loss” and 4 as “stable but inactive.”

Results from a recent hydrogen/deuterium exchange (HDX) study on WT NQO1 (Vankova et al., 2019) enables us to evaluate the local stability of the protein segments in which these residues are found as well as the effect of FAD and dicoumarol binding (two ligands of functional and stability relevance) (Figure 2C). The L7P, L7R and V9I substitutions are located in regions with high stability that do not change upon binding of FAD or dicoumarol (Dic; a competitive inhibitor of NADH). Variants G3S, G3D, A29T, K32N, G34V, E36K, S40L and H162N are located in regions with intermediate HDX stability and whose local stability is hardly sensitive to ligand binding. Nevertheless, these positions could still, in principle, affect protein folding, stability, or solubility and indirectly affect the binding of the substrates. M45L and M45I are found in a region with low stability and not responsive to ligand binding. Y20N is located in a region with intermediate stability and where HDX shows a response to ligand binding; the remaining six variants (T16M, I51V, W106R, W106C, F107C and M155I) are in regions with low stability and also their local stability respond directly to ligand binding. This last group of variants may thus have a greater potential to affect enzymatic activity [preventing either the formation of the “stable” holo-protein, a precatalytic state, or the formation of a catalytically relevant state, the Dic state, with FAD and the inhibitor Dic bound (Anoz-Carbonell et al., 2020)]. Although this suggestion is simple, it must be noted that regions of either high or low local stability may play roles in enzyme functional and allosteric response, and that local effects can propagate far from the perturbed site (due to ligand binding or amino acid substitutions) (Luque and Freire, 2000; Luque et al., 2002; Naganathan, 2019). Therefore, we next performed an experimental characterization of variant effects on protein stability and function and compared the results with our computational analyses.

### 3.3 Variant effects on the expression levels, solubility and stability of NQO1

**FIGURE 3:**
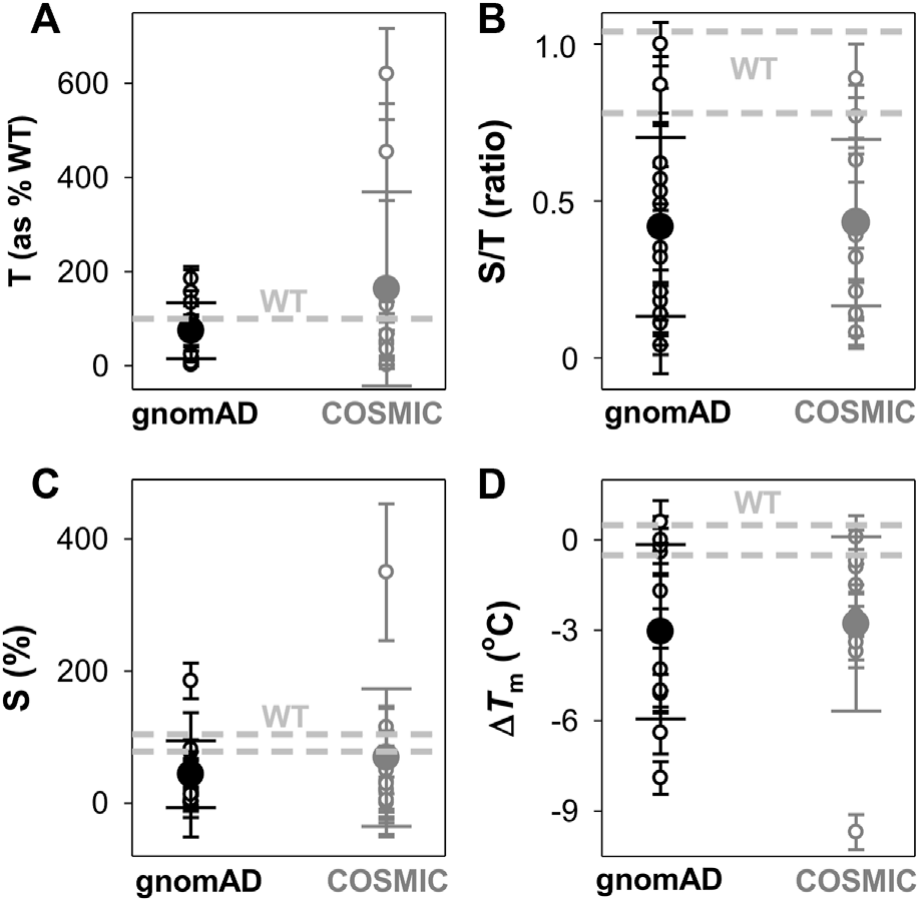
Overall effects of gnomAD and COSMIC variations on the solubility/aggregation propensity and thermal stability of the NQO1 protein. **(A–C)** Expression analyses of solubility/aggregation propensity of NQO1 variants in *E.coli* at 37^o^C. The total amount of NQO1 protein [T, **(A)]** as well as the ratio of soluble/total (S/T) protein **(B)** were determined from induced cells sonicated before (T) and after (S) centrifugation at 21,000 *g*. The levels of NQO1 protein were determined by western-blotting (Supplementary Figure S3). The amount of soluble protein (S) is calculated as the product of T and S/T and shown in **(C)**. Data are the mean ± s.d. from at least three independent expression experiments for each variant; **(D)** Thermal stability of NQO1 variants as holo-proteins. Δ*T*_m_ values correspond to the difference between a given variant and the WT protein. Data are the mean ± s.d. from three-six technical replicas. Small circles indicate the effects of individual variants and large circles (and corresponding errors) are those for each data set (mean ± s.d.). For reference, the values corresponding to WT NQO1 are shown in light grey. Variants are grouped in the gnomAD and COSMIC sets.

**FIGURE 4:**
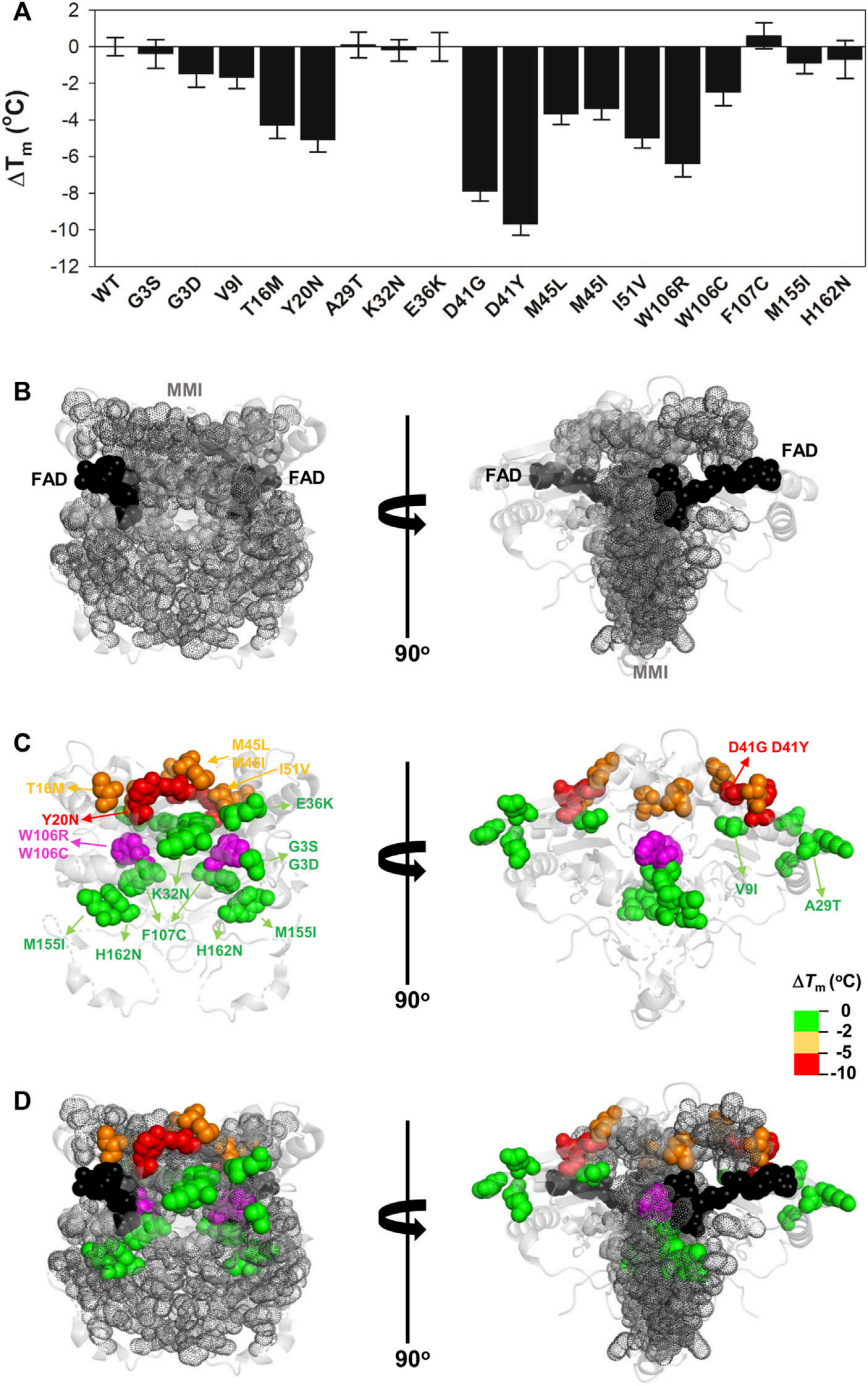
Variant effects on thermal stability related to their location near the MMI or the bound FAD. **(A)** Experimental Δ*T*_m_ values for individual variants; **(B–D)** Structural location of the FAD (black spheres) and the MMI (grey dots) **(B)** and mutated residues (colour scale according to their destabilizing effect) **(C)**. **(D)** shows an overlay of **(B,C)**. Note that two views rotated 90° are shown. The structural model used for display was PDB code 2F1O (Asher et al., 2006). The residue W106 is highlighted in magenta due to the widely different effects of the W106R/W106C substitutions.

We first experimentally analysed the effect of these 22 NQO1 variants on the expression levels and solubility of the protein (upon expression in *E.coli*) as well as their effects on thermal stability (Supplementary Table S1; Figures 3, 4).

The analysis of the total (T) expression of the variants vs. WT NQO1 at 37°C (Figures 3A,B; Supplementary Figure S3; Supplementary Table S1) revealed that some variants (G3S, G3D, L7P, and V9I) showed higher total expression levels, in agreement with our previous report (Pacheco-García et al., 2020). This is likely the result of codon optimization used in the mutagenesis. Most of the variants showed relatively high expression levels, ranging from 25% to full WT levels, indicating that these variants mildly to moderately reduced total expression levels. The L7R, G34V, S40L, D41G, and D41Y variants showed extremely low expression levels, thus preventing further biophysical characterization. Interestingly, overall, no substantial differences were observed between the average effect of the gnomAD and COSMIC sets of variants.

We then determined the fraction of NQO1 existing as soluble protein (S/T ratios; Figure 3B; Supplementary Table S1). WT NQO1 showed a ratio of ∼0.9 (Supplementary Table S1; Figure 3B). Again, although some variants showed much lower S/T ratios than WT NQO1, most of them showed values between 0.2 and the WT ratio. Only five variants showed lower S/T ratios than 0.15 (L7P, L7R, S40L, F107C and M155I). Expression under milder conditions (25°C) did not allow for purification of enough protein of the L7P, L7R, S40L, and G34V variants for further biophysical characterization.

We used the product of total expression levels and S/T ratios (i.e. the S values) as a proxy to evaluate the overall effect of amino acid substitutions on NQO1 solubility/aggregation propensity when expressed at 37°C (Supplementary Table S1; Figure 3C). Nine substitutions reduced the solubility below 20% of the WT protein (L7R, G34V, S40L, D41G, D41Y, M45I, W106R, F107C, and M155I).

Next, we determined the thermal stability of the remaining eighteen variants as holo-proteins (i.e. those that were expressed well as soluble proteins and were stable during purification) (Figures 3D, 4, Supplementary Table S1). Nine variants showed a thermal stability close that of the WT protein (Δ*T*_m_ ≤ 2°C; variants G3S, G3D, V9I, A29T, K32N, E36K, F107C, M155I, and H162N), whereas five variants decreased thermal stability by 2–5°C (variants T16M, M45L, M45I, I51V, and W106C) and four decreased the stability by 5–10°C (Y20N, D41G, D41Y, and W106R) (Supplementary Table S1). Most of the variants that destabilized the holo-protein by more than 2°C are found in the MMI or close to the FAD bound (Figure 4). Here, we note that the reported *T*_m_ and Δ*T*_m_ values are “apparent” values that cannot be regarded as reporting effects on thermodynamic stability since thermal unfolding is irreversible and kinetically-controlled (Pey et al., 2004). The W106R and W106C variants show different effects, highlighting the importance of both the location and the chemical nature of the change. The effects of the two mutations at W106 were intriguing. Both substitutions are non-conservative changes at a residue in the active site with low solvent exposure and a low structural stability with strong ligand-dependent responsiveness based on HDX studies (Vankova et al., 2019). However, their effects are very different, with W106R causing a much larger decrease in stability than W106C.

To end this section, the observed effects pinpoint that some variants in both the COSMIC and gnomAD databases decrease solubility and thermal stability of NQO1, and overall the two groups do so to similar extents (Figure 3); this observation was also seen in the computational predictions (Figure 1).

### 3.4 FAD binding and the stability of the FAD binding site

**FIGURE 5:**
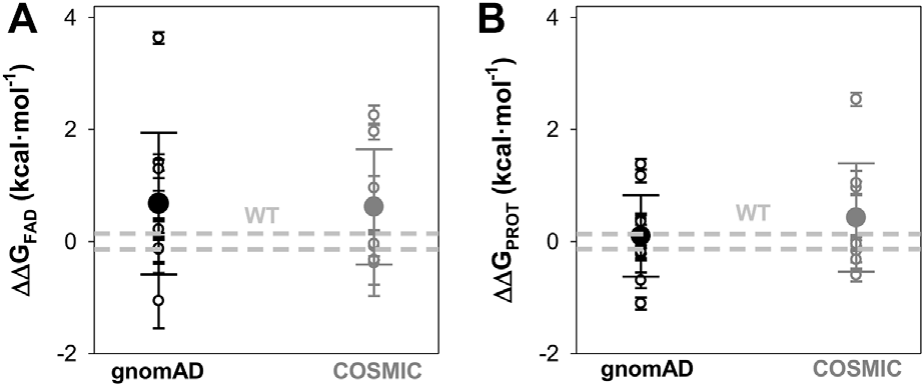
Overall effects of gnomAD and COSMIC mutations on the FAD binding affinity and the local stability of the FAD binding site (FBS). **(A)** Changes in apparent FAD binding free energies (ΔΔG_FAD_) calculated from the difference of binding affinity between WT and a given variant from titrations. Errors are those from linear propagation; **(B)** Local stability of the TCS (next to the FBS) from proteolysis kinetics. Changes in local stability (ΔΔG_PROT_) calculated from the difference of the second-order rate constant between WT and a given variant. Errors are those from linear propagation; For reference, the values corresponding to WT NQO1 are shown in light grey. Variants are grouped in the gnomAD and COSMIC sets.

**FIGURE 6:**
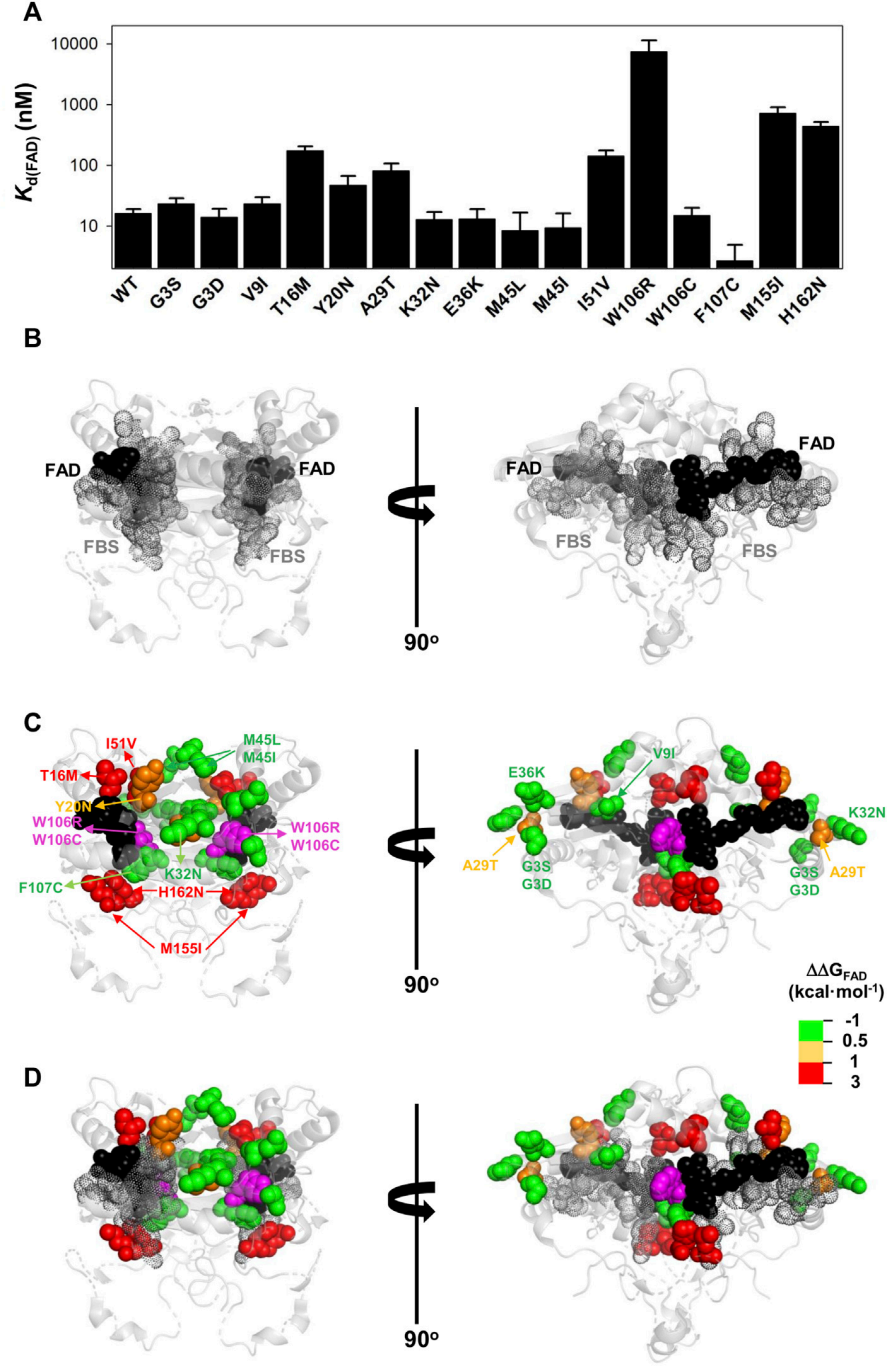
Variant effects on the FAD binding affinity. **(A)** FAD binding affinity of each variant was determined by titrations of apo-proteins. At least two independent experiments were carried out for each variant and fitted using a single-type-of-independent binding sites to obtain *K*_d_ values (note the logarithmic scale of the *y*-axis). Errors are those fittings. These *K*_d_ values were used to calculate the binding free energy difference (ΔΔG_FAD_) between a given variant and the WT protein (note that a positive value indicates lower affinity). **(B–D)** Structural location of the FAD (black spheres) and the FBS (grey dots) **(B)** and mutated residues (colour scale according to their destabilizing effect on FAD binding) **(C)**. The residue W106 is highlighted in magenta due to the widely different effects of the W106R/W106C substitutions. **(D)** shows an overlay of **(B,C)**. Note that two views rotated 90° are shown. The structural model used for display was PDB code 2F1O (Asher et al., 2006).

Sixteen out of the eighteen variants that yielded good levels of soluble proteins were prepared as apo-proteins to determine their affinity for FAD by titrations monitored by tryptophan fluorescence (Figures 5A, 6; Supplementary Figure S4; Supplementary Table S2). The D41Y and D41G variants were too unstable to obtain apo-proteins in sufficient amounts and quality.

The G3S, G3D, V9I, K32N, E36K, M45L, M45I, W106C, and F107C variants showed less than a 0.5 kcal mol^−1^ increase in FAD binding free energy (corresponding to less than a 2.5-fold increase in *K*_d_). The Y20N and A29T variants showed a moderate decrease in binding affinity (between 3 and 5-fold higher *K*_d_; thus, a change in FAD binding free energy of 0.5–1.0 kcal mol^−1^). Five variants (T16M, I51V, W106R, M155I and H162N) markedly decreased the binding affinity for FAD (10–500-fold increase in *K*_d_; between 1 and 3.7 kcal mol^−1^ decrease in binding free energy). Inspection of a structural model of NQO1 allows us to rationalize the effect of these substitutions due to their proximity to the bound FAD, with some exceptions. For instance, the W106R, W106C and F107C substitutions are in proximity to the FAD molecule, but have widely different effects (from ca. 500-fold lower affinity in W106R, to WT-like affinity for W106C and even *higher* affinity than WT for F107C). These results show that NQO1 responds to natural variations very differently even at the same site (i.e. two highly non-conservative variants at the site W106).

**FIGURE 7:**
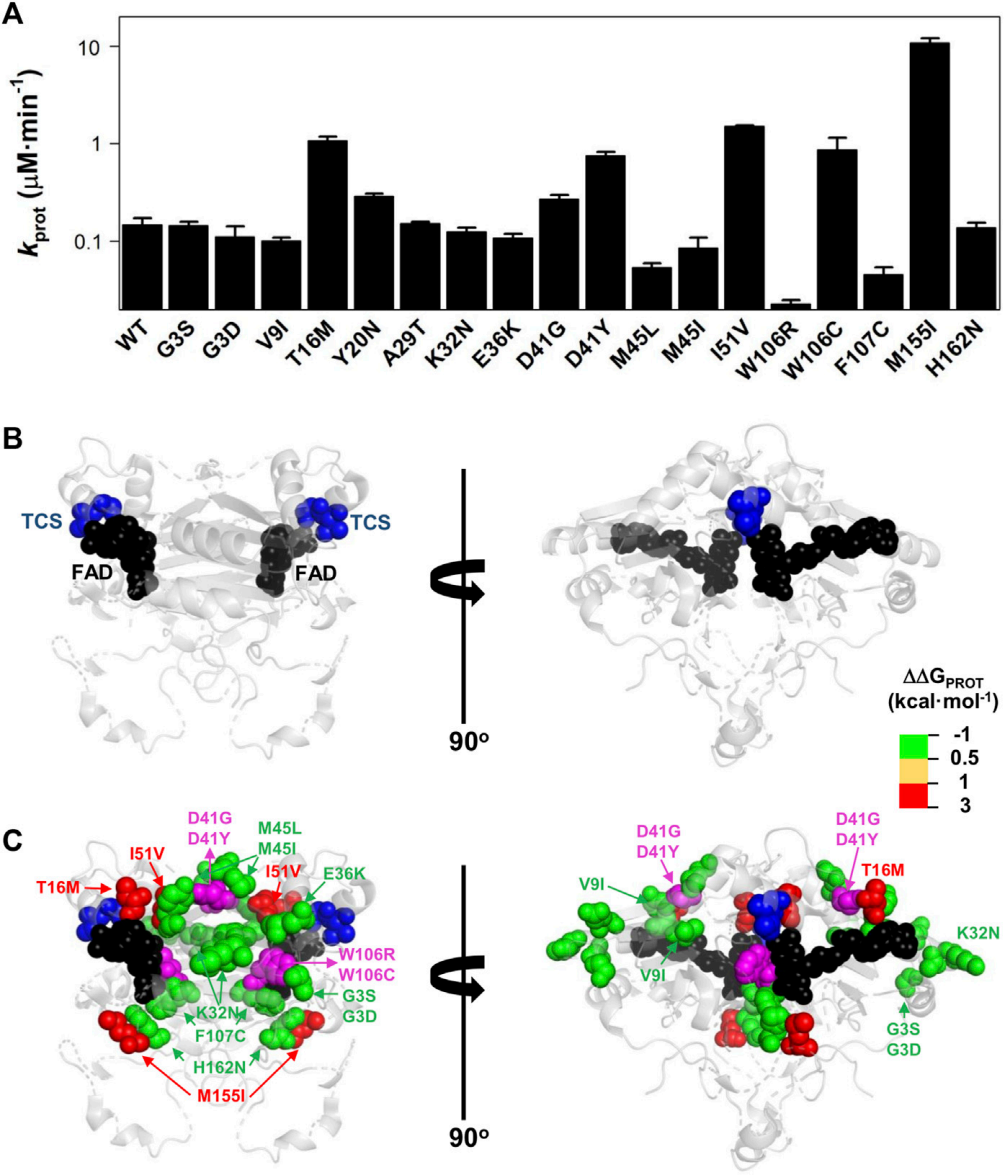
Variant effects on the local stability of the FBS from proteolysis. **(A)** Second-order rate constants for proteolysis of NQO1 variants (Supplementary Figure S5C). Errors are those fittings. These *k*_PROT_ values were used to calculate the TCS local energy free difference (ΔΔG_PROT_) between a given variant and the WT protein (note that a positive value indicates lower affinity). **(B,C)** Structural location of the FAD (black spheres) and the TCS (blue spheres) **(B)**. In **(C)**, mutated residues (colour scale according to their destabilizing effect on FAD binding) **(C)** are overlayed with TCS and FAD. The residues D41 and W106 are highlighted in magenta due to the widely different effects of the D41G/D41Y and W106R/W106C substitutions. Note that two views rotated 90° are shown. The structural model used for display was PDB code 2F1O (Asher et al., 2006).

Mutational effects on FAD binding affinity (ΔΔG_FAD_) and proteolysis rates (ΔΔG_PROT_) with thermolysin have previously been shown to correlate well (Medina-Carmona et al., 2017a; Medina-Carmona et al., 2019b; Pacheco-García et al., 2020). The site for initial cleavage by thermolysin (TCS) is generally between Ser72–Val73, close to the FAD binding site (Medina-Carmona et al., 2016). All the variants investigated showed proteolysis patterns that were similar to that of WT NQO1 (Supplementary Figure S5). The previously observed correlation between ΔΔG_FAD_ and ΔΔG_PROT_ holds for the 16 variants for which both FAD binding affinity and protease sensitivity can be compared (Supplementary Figure S6). The T16M, I51V, W106C, and M155I mutations destabilized locally the TCS by 1–2.5 kcal mol^−1^ and the residues affected by these substitutions are in general close to the TCS (with the exception of M155I, the most destabilizing mutation) (Figure 7). Again, the results for the W106R/C variants were very different: both affected the local stability by ∼ 1 kcal mol^−1^, but with opposite signs (Supplementary Table S2).

Overall, the negative impact on FAD binding affinity and the stability of the FAD binding site in the holo-state was similar between variants from COSMIC and gnomAD sets (Figure 5).

### 3.5 Comparison of experimental analyses and computational predictions

**FIGURE 8:**
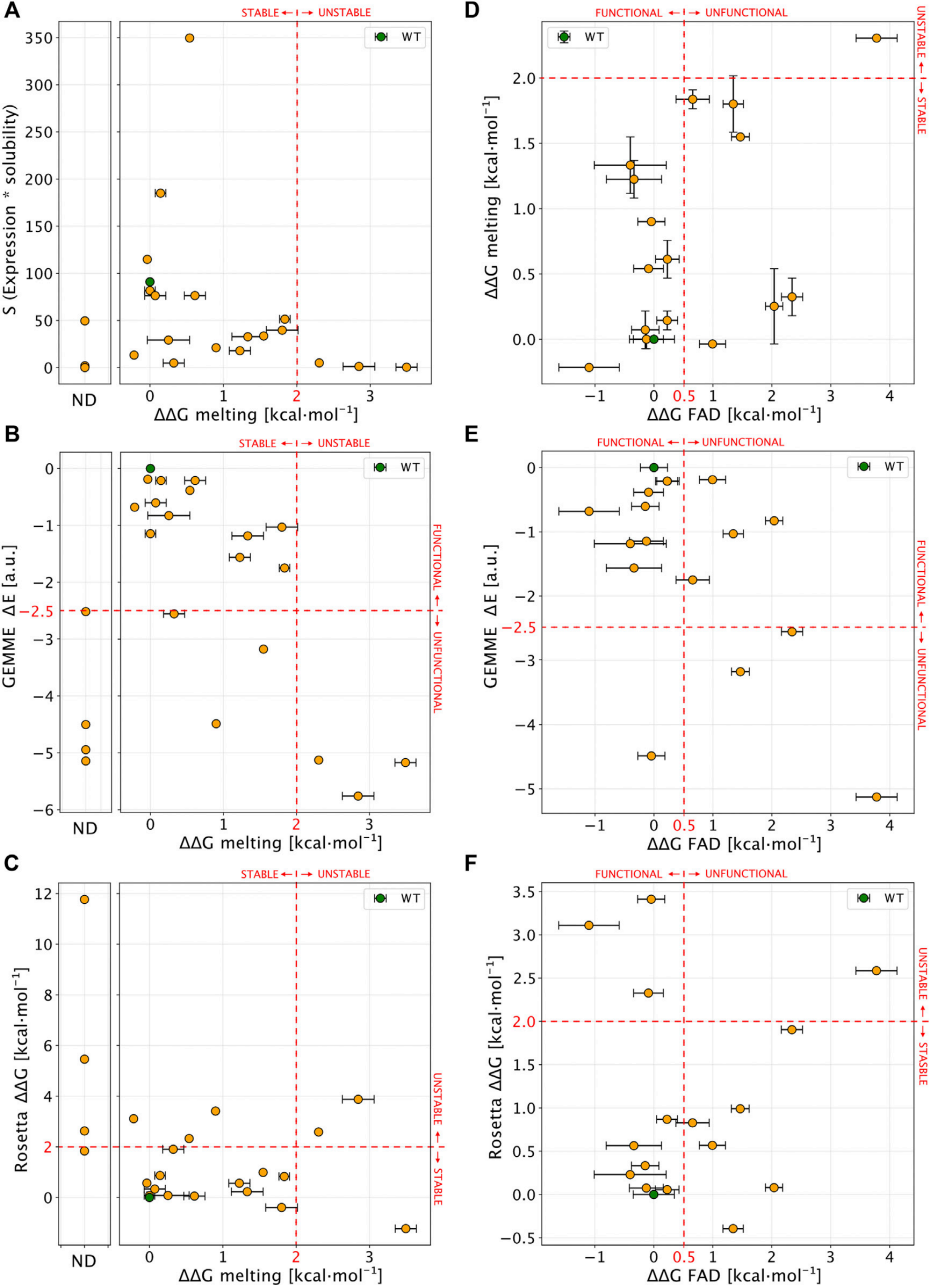
Comparison of experimental results with computational scores. **(A)** Scatter plot of ΔΔG_melting_ (*x*-axis) and S values (expression * solubility). Not-determined variants (ND) in thermal stability experiment are reported in a separate plot. **(B,C)** show a comparison between ΔΔG_melting_ and computational predictors. **(D)** Correlation between ΔΔG_melting_ and ΔΔG_FAD_ for NQO1 variants detected in both the experiments. **(E,F)** Comparison of ΔΔG_FAD_ with the computational results. Red lines, if present, show the boundary of experimental and computational classes for each comparison. In each panel errors are reported as a black bar on every single variant, if present.

We then proceeded to compare the experimental data to each other and to computational predictions. To ease comparison between the calculated ΔΔG values and thermal melting measurements, we first estimated ΔΔG_melting_ from the Δ*T*_m_ values using an empirical relationship (Watson et al., 2018), again noting that these are not strictly experimental thermodynamic values as unfolding is not reversible either by temperature (Pey et al., 2004) or chemical denaturants (Figure S7). We first compared the experimental values of ΔΔG_melting_ to the levels of soluble protein (S values, Figure 8A). We found that unstable variants (those with ΔΔG_melting_ > 2 kcal mol^−1^ or not amenable for purification, not-determined or ND) mostly showed level of S close to zero (<5) except for L7P. Stable variants (here defined as ΔΔG_melting_ < 2 kcal mol^−1^) instead showed a wide range of S values (76 ± 85%; mean ± s.d.).

We next compared ΔΔG_melting_ with the computational scores (Figures 8B,C). Overall, we found a good agreement for most of the unstable and Not-Determined (ND) variants, which showed ΔΔG >2.0 kcal mol^−1^ and evolutionary distance, ΔE < −3 indicating predicted loss of stability and function. The only exception was D41Y which displayed a stabilizing behaviour in Rosetta ΔΔG predictions. Experimentally stable variants (ΔΔG_melting_ < 2 kcal mol^−1^) displayed low evolutionary distances (ΔE > −2.5 kcal mol^−1^) except for W106C and T16M. This observation for T16M supports the notion that detrimental effects on protein function may not be connected to thermodynamic destabilization for this variant (Pacheco-García et al., 2020).

We then compared ΔΔG_FAD_ with the other experimental and computational observables (Figures 8D–F). For most (15 out of 16) of the variants where ΔΔG_FAD_ could be measured, the ΔΔG_melting_ was <2 kcal mol^−1^, indicating stable variants which was also confirmed by Rosetta ΔΔG calculations (13 out of 16 substitutions). Seven of the fifteen variants showed a ΔΔG_FAD_ > 0.5 kcal mol^−1^ indicating loss of function. Of these, three variants were captured by evolutionary conservation analysis (ΔE < −2.5 kcal mol^−1^).

To summarize, for 14 out of 22 tested variants (G3S L7P L7D V9I T16M Y20N K32N G34V E36K D41G M45L M45I W106R, M155I) the predictions from the computational protocols match the experimental results, in terms of variant effects on protein stability and function.

While the results are overall encouraging, there remains differences between computation and experiments for the effects of some mutations (eight out of twenty-two; G3D, A29T, S40L, D41Y, I51V, W106C, F107C, and H162N). For five of these (G3D, S40L, D41Y, W106C, and F107C) it appears that there is a difference between the stability prediction by Rosetta and experiments (noting again that the latter are not equilibrium measurements). For the partially exposed S40L and D41Y the reason for the discrepancy is perhaps related to specific interactions made by these two residues whose effects are not captured by the Rosetta calculations. Both W106C and F107C involve substituting aromatic residues with a cysteine, suggesting problems with evaluating such substitutions. In other two cases (G3D, A29T, and H162N) the GEMME scores did not capture properly the variant effects, possibly because some specific interactions in human NQO1 may not be present in other homologs of NQO1 and thus, not captured by the evolutionary analysis. Lastly, for I51V the behaviour is opposite from both computational predictions.

## 4 Discussion

With advances in sequencing technologies, we are uncovering the large genetic variability in the *human genome*. To exploit the availability of this information at the clinical level, we must be able to establish genotype-phenotype correlations accurately and at a large-scale. Although detailed characterization of mutational effects is obviously useful, it is difficult to perform this at such scale (many genes, many variants). However, we may use experimental characterization on a more modest scale to test the performance of current predictive tools in the hope that we can improve them. In this work, we have carried out such an exercise for the human NQO1 protein. The rationale for selecting this system is three-fold: 1) human NQO1 is a multifunctional protein and mutational effects may affect these functions through complex mechanisms (Pacheco-Garcia et al., 2022a; Pacheco-Garcia et al., 2022b). Therefore, contrasting experimental characterization and computational predictions can be challenging for current predictive tools and may help to improve them; 2) Altered NQO1 functionality is associated with increased risk of developing cancer and neurological disorders (Salido et al., 2022). Indeed, the presence of a highly deleterious polymorphic variant in NQO1 is associated with increased cancer risk and affects multiple protein functions through allosteric effects (Lajin and Alachkar, 2013; Pacheco-Garcia et al., 2022b); 3) Over a hundred of missense variants in human NQO1 have been found in human population (i.e. the gnomAD database) or in cancer cell lines as somatic mutations (i.e. the COSMIC database). However, the impact of these mutations on NQO1 multifunctionality and their potential role in cancer development are largely unexplored.

Theoretical advances and new methodologies in the fields of sequence evolution and structure predictions allow us to perform large-scale *in silico* mutagenesis studies on target proteins. Although the current state-of-art algorithms are often [but not always (Frazer et al., 2021)] less accurate at predicting pathogenicity compared to detailed experimental testing, they provide a fast and effective way to predict LoF and sometimes to generate mechanistic hypotheses regarding which properties a variant might affect (Stein et al., 2019; Cagiada et al., 2021).

Here, we first performed *in silico* saturation mutagenesis of WT NQO1, predicting the changes in thermodynamic stability (ΔΔG) and evolutionary conservation (ΔE) for 5,187 variants. We combined the two scores to perform a global analysis on how the NQO1 function may be perturbed. Approximately 44% of variants are predicted to cause LoF, with 45% of these drastically affecting the protein stability. This analysis enabled us to obtain an overview on the possible biologically relevant positions and variants. Indeed, although we know that the ability of computational tools to assign biological functions and predict overall pathogenicity is rapidly increasing, we are still at a point where computational methods may not predict LoF perfectly, and often do not shed much light on the mechanisms of action. This might in particular hold for proteins like NQO1 where multiple biological functions are present, and where some of which may differ between orthologues.

We then used the information provided from the *in silico* saturation mutagenesis to select 22 naturally-occurring variants with a diverse range of predicted effects on protein stability and function to be experimentally tested. We selected nine mutations found in COSMIC and thirteen from gnomAD. Of these variants, 36% severely affected protein foldability and solubility (upon expression in *E.coli*) or reduced conformational stability (at least a 5°C decrease in *T*_m_). A quarter of the variants had severely affected FAD binding (a 5-fold decrease in affinity, i.e. a 1 kcal mol^−1^ of binding free energy penalization). For 64% of the variants, experimental characterization and computational predictions agreed in the variant effects on protein stability and function, whereas the remaining 36% of the mutations might be explained by limitations known for the tools used in the prediction process. Although, at this point, this level of agreement is reasonable, it also pinpoints the necessity of improving these predictive tools.

COSMIC mutations are in general somatic (actually, 86% of the COSMIC mutations of NQO1 are labelled as *confirmed* in this database; accessed by 17 August 2022) and likely come from samples that underwent many mutational events in different genes. Thus, the identification of a mutation in the COSMIC database does not imply that this mutation is a driver mutation [here we may define a driver mutation as a mutation with the ability to drive tumorigenesis and confer selective advantages in a tumor cell and a somatic tissue (Martínez-Jiménez et al., 2020)]. Mutations in the gnomAD database belong to heterogeneous groups (many different sequencing projects, some of them case-control studies), and likely reflect genetic variability in the *germline* and in general presence or absence in gnomAD is not sufficient to assign a label as pathogenic or benign. When we examine the NQO1 variants investigated in this work found in the gnomAD database (v.2.1), allele frequencies are overall comparable in *control* vs. *all* samples (Supplementary Table S3). This suggests that there is no strong bias towards *case* samples, and thus the allele frequencies in gnomAD may represent well their presence in a *healthy* population. The presence of these mutations in the germline may predispose somatic cells towards a new mutational event in the WT allele [as occurs in familial cancer cases (Martínez-Jiménez et al., 2020)], thus largely decreasing the NQO1 activity and function.

Our combined experimental and computational analyses provide information on the potential LoF character and the mechanisms by which the variants may exert their effects (protein stability and/or function). Due to its role in the antioxidant defense and stabilization of oncosuppressor proteins, it is likely that NQO1 play a role in cancer development. Homozygous NQO1 knock-out mice revealed cancer-associated phenotypic traits when exposed to chemical or radiological insults (Radjendirane et al., 1998; Long et al., 2000; Iskander et al., 2005; Iskander et al., 2008). Thus, the presence of LoF variants in NQO1 and increased cancer risk may resemble a recessive inheritance (Lajin and Alachkar, 2013). The p.P187S polymorphism (with an allele frequency of ∼0.25, Supplementary Table S3) dramatically decreases the intracellular stability of NQO1 thus preventing its interaction with oncosuppressors, reducing enzyme activity and affecting almost the entire structure of NQO1 (Pacheco-Garcia et al., 2021). Noteworthy, it only associates with cancer in homozygotes (Lajin and Alachkar, 2013). Due to the low frequency of most gnomAD NQO1 variants, their presence would be rare even in compound heterozygotes. In fact, 98% of the homozygous samples containing NQO1 missense variations correspond to homozygotes for P187S. However, an additional (*somatic*) mutational event in a WT/P187S genetic background (about 25% of human population) may substantially enhance the LOF phenotype in this common genetic background.

To conclude, we present a test of predictive tools against the experimental characterization of large set of naturally-occurring mutations on NQO1 stability and function. Further steps will be taken to provide a wider perspective on the multifunctionality of NQO1 (i.e. intracellular degradation and stability, high-resolution structural stability in different ligation states, enzyme function and cooperativity, interaction with protein partners, allosteric communication of mutational effects) and the relationships between the genetic diversity of NQO1 in human population and its link with individual propensity towards disease development.

## Data Availability

The original contributions presented in the study are included in the article/Supplementary Material, further inquiries can be directed to the corresponding author.

## Abbreviations

CTD, C-terminal domain; COSMIC, Catalogue Of Somatic Mutations In Cancer cell lines database; Dic, dicoumarol; FAD, Flavin adenine dinucleotide; GEMME: Global Epistatic Model for predicting Mutational Effects; gnomAD, Genome Aggregation Database; HDX, hydrogen/deuterium exchange; HEPES, 2-[4-(2-hydroxyethyl)piperazin-1-yl]ethanesulfonic acid; IMAC, Immobilized metal affinity chromatography; *K*_d_, apparent dissociation constant; *k*_obs_, observed rate constant for proteolysis; *k*_prot_, second-order rate constant for proteolysis; LoF, Loss-of-function; MMI, monomer:monomer interface; MSA, multiple sequence alignment; NTD, N-terminal domain; NQO1, NAD(P)H:quinone oxidoreductase 1; S, soluble protein levels; SASA, solvent accessible surface area; SDS-PAGE, polyacrylamide gel electrophoresis in the presence of sodium dodecylsulphate; S/T, Soluble/Total ratio of NQO1 protein; *T*_m_, apparent half-denaturation temperature; WT, wild-type; Δ*E*, Normalized predicted effect of an amino acid substitution from the GEMME model; ΔΔ*G*, computationally calculated change in unfolding Gibbs free energy between a variant and the WT protein; ΔΔ*G*_FAD_, difference in binding Gibbs free energy between a variant and the WT protein; ΔΔ*G*_melting_, operational metric for the estimation of the unfolding Gibbs free energy changes between a variant and the WT protein from Δ*T*_m_; ΔΔ*G*_PROT_, difference in free energy of cleavable and non-cleavable states for proteolysis between a variant and the WT protein; Δ*T*_m_, difference in *T*_m_ between a variant and the WT protein.
