# Supplementary material for: Effect of naturally-occurring mutations on the stability and function of cancer-associated NQO1: Comparison of experiments and computation
Source: Front Mol Biosci. 2022 Nov 24;9:1063620. doi: 10.3389/fmolb.2022.1063620 (PMC9730889; doi:10.3389/fmolb.2022.1063620)
Supplement: Supplementary file 1 [file Presentation1.zip › Suppl. Figure 1.DOCX]

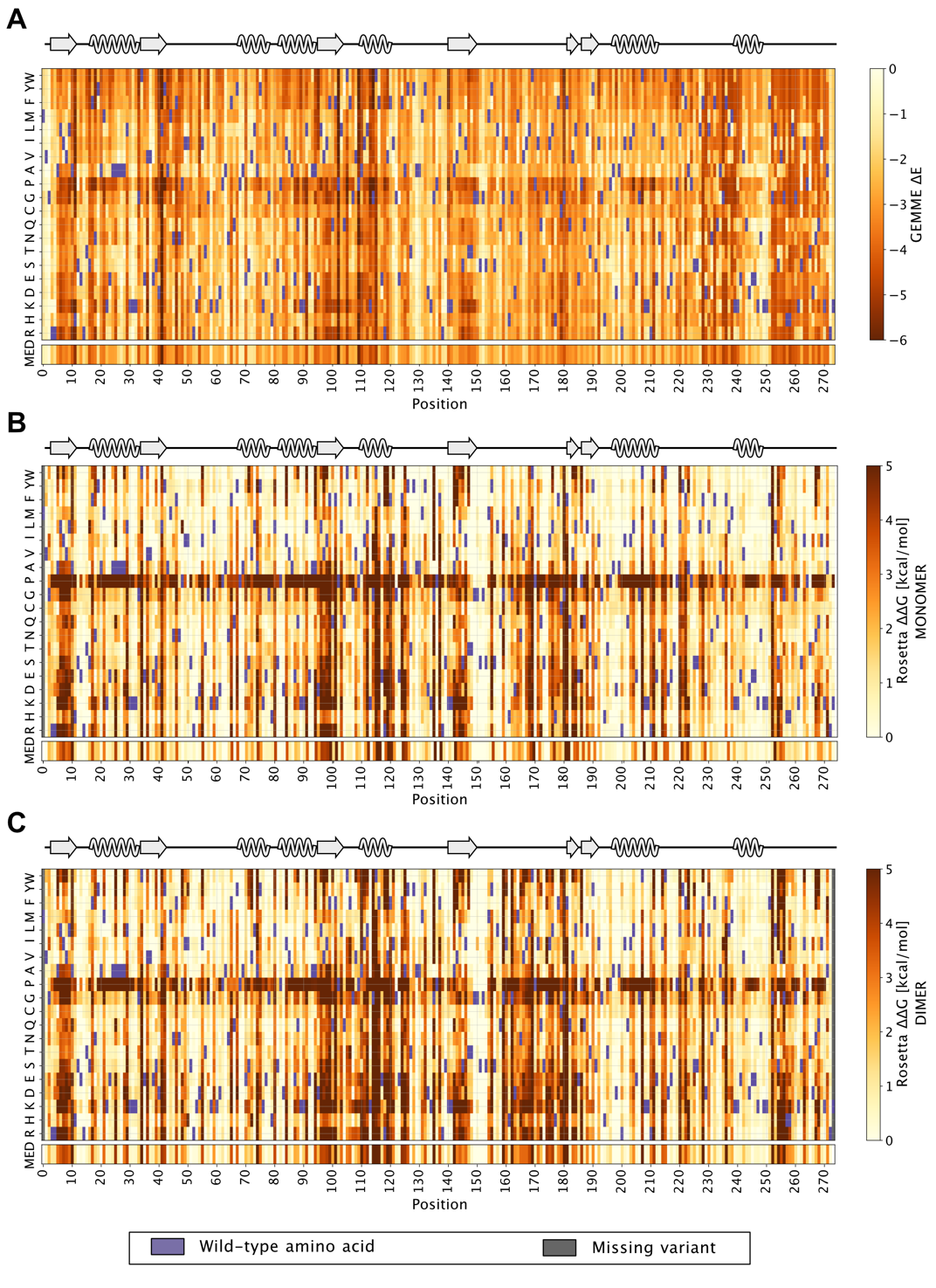


**Supplementary Figure 1. Computational prediction maps of thermodynamic stability and evolutionary conservation of NQO1.** Panel A shows the results of evolutionary conservation analysis using GEMME on NQO1 sequence. Variants evolutionary close to the WT have score close to zero, while variants with detrimental effects on the protein have high negative scores (red shadows). WT amino acid is indicated with a purple box and the median score is shown for each residue. Panel B and C show the thermodynamic stability (ΔΔG) maps of NQO1 evaluated using Rosetta using the monomer (B) and the dimer (C) structure. Variants with a stability similar to the wild-type have scores close to zero, while variants with detrimental effects on the protein’s stability have large positive scores (coloured with shadows of red). Wild-type amino acids are indicated with purple boxed, positions (and therefore variants) which are missed in the PDB structure used are coloured in grey and the median score is shown for each position.
