# Supplementary material for: Effect of naturally-occurring mutations on the stability and function of cancer-associated NQO1: Comparison of experiments and computation
Source: Front Mol Biosci. 2022 Nov 24;9:1063620. doi: 10.3389/fmolb.2022.1063620 (PMC9730889; doi:10.3389/fmolb.2022.1063620)
Supplement: Supplementary file 1 [file Presentation1.zip › Suppl. Figure 2.DOCX]

**
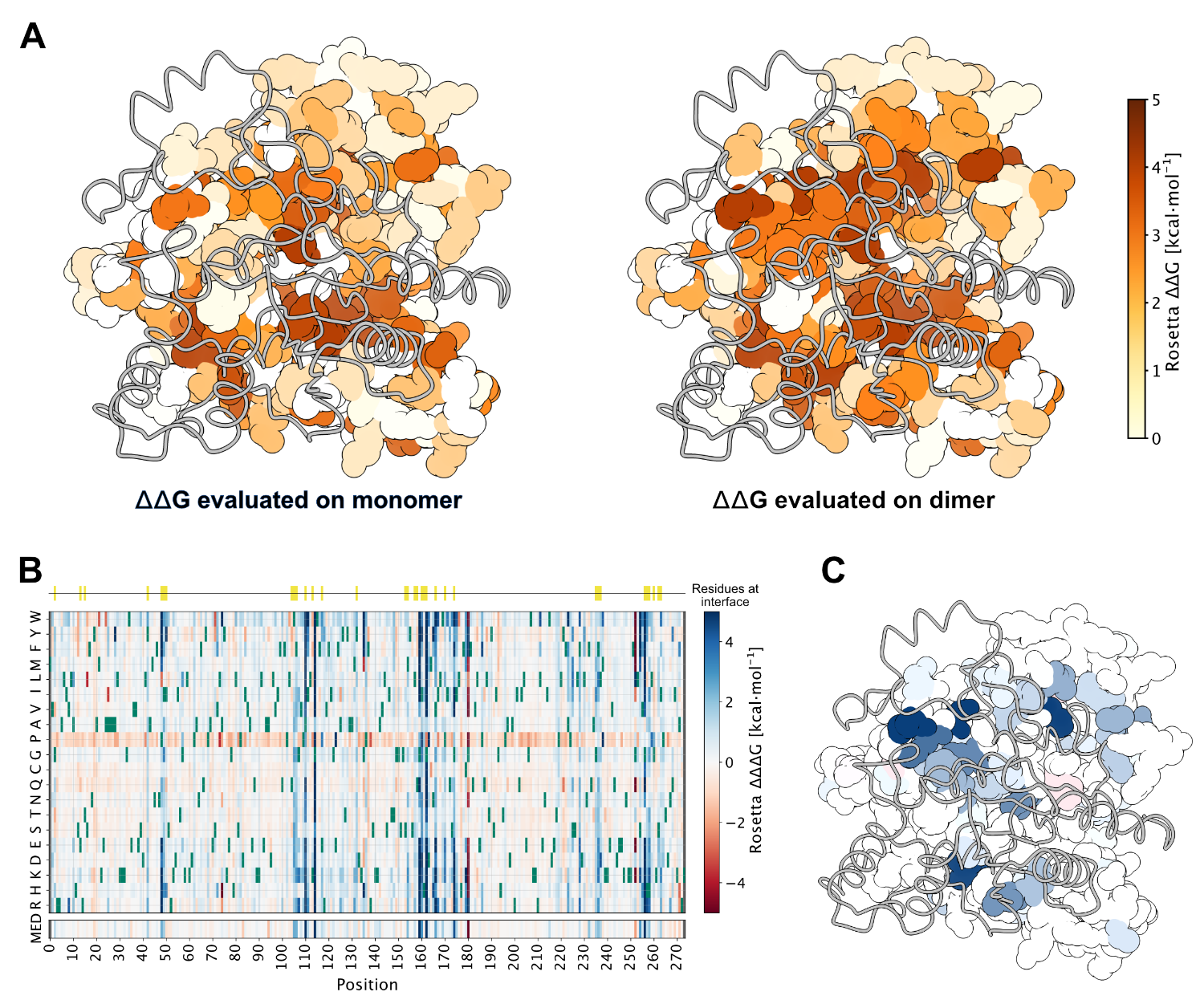
**

**Supplementary Figure 2.** **Thermodynamic stability differences (ΔΔG) between the monomer and dimer based on computational methods.** Panel A shows the median ΔΔG value for each residue on the crystal structure of NQO1, the figure on the left show median ΔΔGs evaluated keeping only the monomer structure of the NQO1 while the figure on the right report median ΔΔG evaluation using homozygous mutation on the dimer. Positions with neutral effect are coloured in white, while detrimental positions are coloured in shadows of red. Panel B shows the heatmap with the Δ(ΔΔG) between the two different ΔΔG evaluations on the monomer and dimer. Variants with a higher stability in dimer evaluation of ΔΔG are coloured in blue while variants with a higher stability in the monomer ΔΔG are coloured in red. WT residues are reported in green. On the top of the heatmap, interface residues are reported with a yellow marker. Panel C shows the median positional Δ(ΔΔG) difference between the two evaluations mapped to the crystal structure of NQO1.
