# Supplementary material for: Effect of naturally-occurring mutations on the stability and function of cancer-associated NQO1: Comparison of experiments and computation
Source: Front Mol Biosci. 2022 Nov 24;9:1063620. doi: 10.3389/fmolb.2022.1063620 (PMC9730889; doi:10.3389/fmolb.2022.1063620)
Supplement: Supplementary file 1 [file Presentation1.zip › Suppl. Figure 3.DOCX]

**
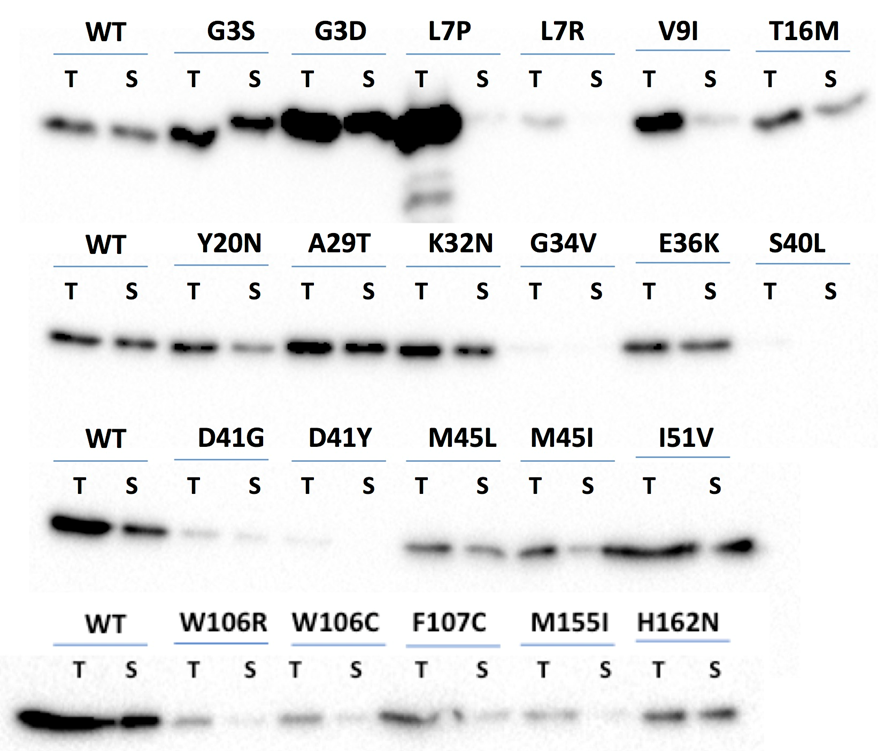
**

**Supplementary Figure 3. Representative Western-blot analyses of total (T) and soluble (S) expression levels for NQO1 variants in *E.coli*.** Experimental details can be found in the main text
