# Supplementary material for: Effect of naturally-occurring mutations on the stability and function of cancer-associated NQO1: Comparison of experiments and computation
Source: Front Mol Biosci. 2022 Nov 24;9:1063620. doi: 10.3389/fmolb.2022.1063620 (PMC9730889; doi:10.3389/fmolb.2022.1063620)
Supplement: Supplementary file 1 [file Presentation1.zip › Suppl. Figure 4.DOCX]

**
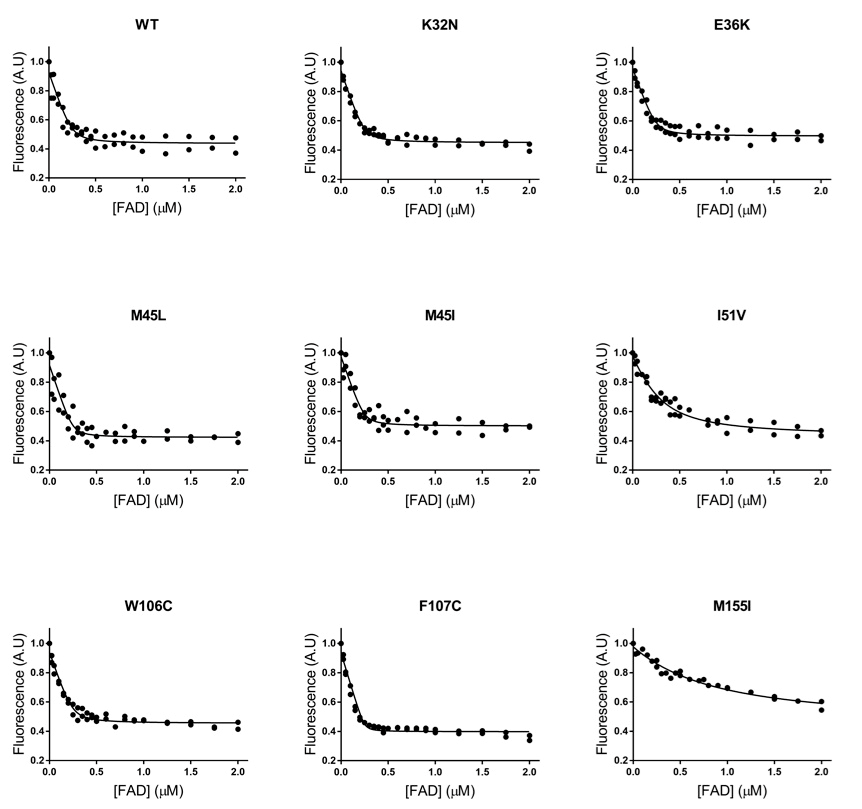
**

**Supplementary Figure 4. Titrations of apo-NQO1 proteins with FAD.** Experiments were replicated twice and all data were fitted to single-site binding model. In all the cases, titrations were carried out by fluorescence measurements. Experimental details can be found in the main text.
