# Supplementary material for: Effect of naturally-occurring mutations on the stability and function of cancer-associated NQO1: Comparison of experiments and computation
Source: Front Mol Biosci. 2022 Nov 24;9:1063620. doi: 10.3389/fmolb.2022.1063620 (PMC9730889; doi:10.3389/fmolb.2022.1063620)
Supplement: Supplementary file 1 [file Presentation1.zip › Suppl. Figure 5.DOCX]

**
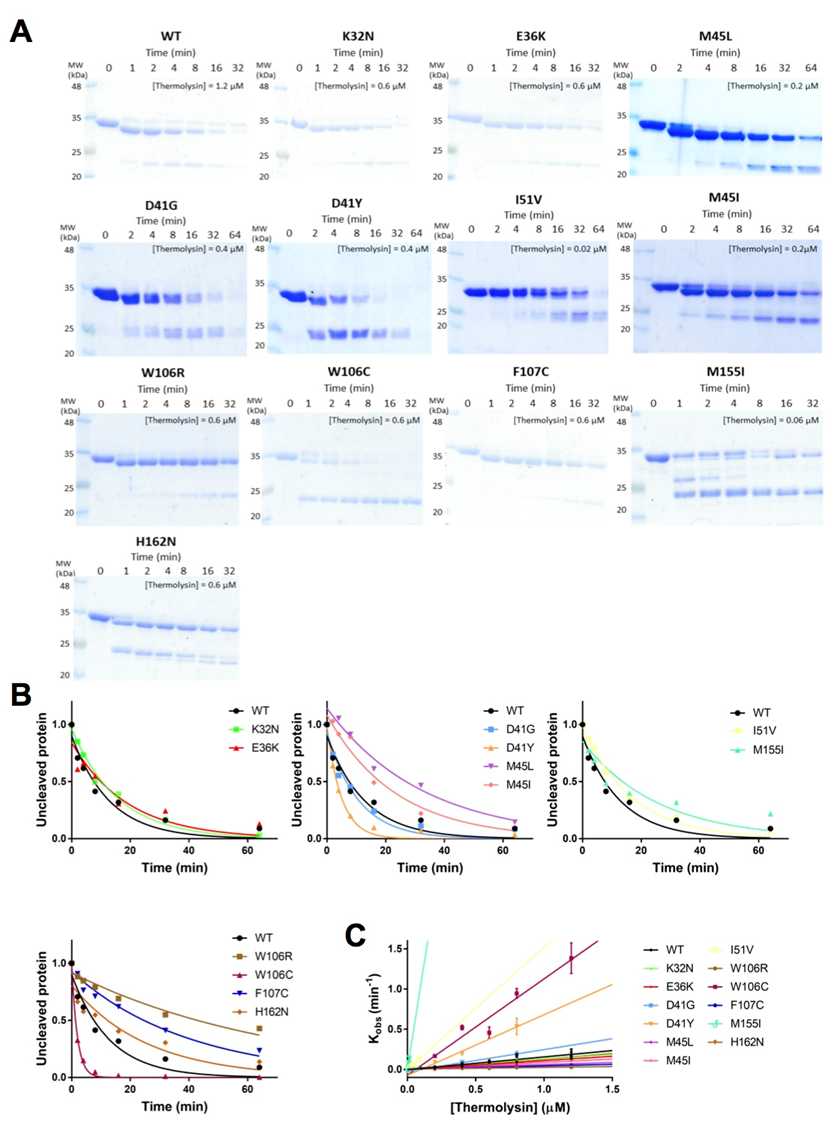
**

**Supplementary Figure 5. Partial proteolysis of NQO1 variants by thermolysin.** Panel A shows representative kinetic experiments with the protease and NQO1 variants. The concentration of protease used is indicated in each case. Panel B shows the corresponding densitometric analysis of native protein over time used to obtained the observed first-order rate constant using 0.4 µM thermolysin. Panel C shows the linear dependence of the observed rate constants that can be interpreted as effects on the thermodynamic stability of the TCS. Experimental details can be found in the main text.
