# Supplementary material for: Effect of naturally-occurring mutations on the stability and function of cancer-associated NQO1: Comparison of experiments and computation
Source: Front Mol Biosci. 2022 Nov 24;9:1063620. doi: 10.3389/fmolb.2022.1063620 (PMC9730889; doi:10.3389/fmolb.2022.1063620)
Supplement: Supplementary file 1 [file Presentation1.zip › Suppl. Figure 6.DOCX]

**
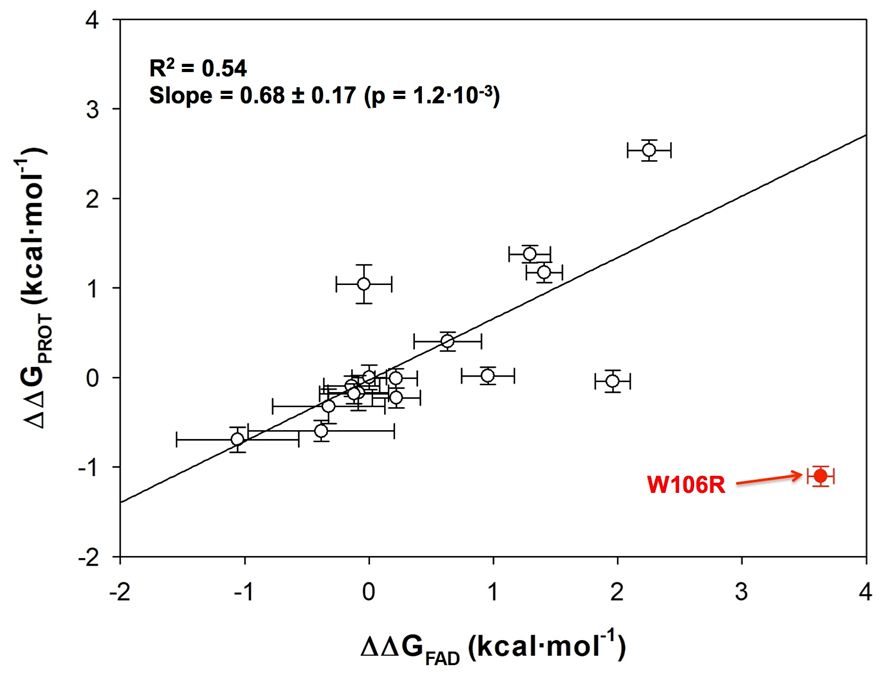
**

**Supplementary Figure 6. Correlation between changes in free energy for limited proteolysis (ΔΔG_PROT_) at the NTD and binding affinity for FAD (ΔΔG_FAD_).** The plot includes the best linear correlation between both parameters. W106R was excluded as a clear outlier.
