# Supplementary material for: Effect of naturally-occurring mutations on the stability and function of cancer-associated NQO1: Comparison of experiments and computation
Source: Front Mol Biosci. 2022 Nov 24;9:1063620. doi: 10.3389/fmolb.2022.1063620 (PMC9730889; doi:10.3389/fmolb.2022.1063620)
Supplement: Supplementary file 1 [file Presentation1.zip › Suppl. Figure 7.DOCX]

**Supplementary Figure 7. Urea induced denaturation of holo-NQO1 WT is not reversible.** A) Fluorescence spectra of hNQO1 in unfolding (black symbols) and refolding (red symbols) experiments. B) the fluorescence intensities at 350 nm in unfolding (black symbols) and refolding (red symbols) experiments. C) λ_max_ of fluorescence emission spectra in unfolding (black symbols) and refolding (red symbols) experiments. For denaturation experiments, 2 µM holo-NQO1 protein was incubated with 2 µM FAD in HEPES-KOH 50 mM, pH 7.4 and 2 mM β-mercaptoethanol in the presence of 0-7.2 M urea for 16 h at 25 ^o^C in the darkness. Denaturation was followed by intrinsic fluorescence (excitation, 280 nm; emission, 320-380 nm; slits 5 nm) at 25 ^o^C. using a Cary Eclipse spectrofluorimeter (Agilent Technologies, Waldbronn, Germany) and a 3 x 3 mm quartz cuvette. Four scans were measured for each sample and averaged. Blanks in the absence of protein were acquired and subtracted. For renaturation experiments, a sample of 20 µM holo-NQO1 protein was incubated with 20 µM FAD in HEPES-KOH 50 mM, pH 7.4 and 2 mM β-mercaptoethanol in the presence of 7.2 M urea for 16 h at 25 ^o^C in the darkness. Then, protein samples were diluted 10-fold to different urea solutions in the range 0.8-6.4 M concentration in HEPES-KOH 50 mM, pH 7.4 and 2 mM β-mercaptoethanol and allowed to “renature” for 6 h at 25 ^o^C before fluorescence spectra were acquired at the same conditions than those described in denaturation experiments. In all cases, urea concentration was determined by refractive index measurements.
