# Supplementary material for: Effect of naturally-occurring mutations on the stability and function of cancer-associated NQO1: Comparison of experiments and computation
Source: Front Mol Biosci. 2022 Nov 24;9:1063620. doi: 10.3389/fmolb.2022.1063620 (PMC9730889; doi:10.3389/fmolb.2022.1063620)
Supplement: Supplementary file 2 [file Table1.DOCX]

**Supplementary Table 1. Variant effects on the solubility and thermal stability of NQO1.** Expression analyses of NQO1 were carried out in *E.coli* at 37^o^C and the total expression levels (T), and the fraction of soluble protein (S/T ratio) determined by western-blot (mean ± s.d. from three independent experiments). The % of soluble protein (S) was thus calculated as the T x S/T product. *T*_m_ values were determined upon purification of holo-NQO1 proteins by thermal denaturation (mean±s.d. from at least three replicates). N.Det. not determined due to very low expression levels as soluble protein.

| **Variant** | **T**  **(% vs.WT)** | **S/T ratio** | **S**  **(%)** | ***T*_m_ (^o^C)** |
| --- | --- | --- | --- | --- |
| WT | 100 | 0.91±0.13 | 91±13 | 51.8±0.5 |
| G3S | 185±26 | 1.00±0.07 | 185±27 | 51.4±0.6* |
| G3D | 454±103 | 0.77±0.10 | 350±103 | 50.3±0.5* |
| L7P | 620±97 | 0.08±0.04 | 50±10 | N.Det. |
| L7R | 9±10 | 0.11±0.03 | ~ 1 | N.Det. |
| V9I | 156±48 | 0.49±0.37 | 76±60 | 50.1±0.3* |
| T16M | 96±10 | 0.35±0.12 | 34±16 | 47.5±0.5* |
| Y20N | 97±10 | 0.53±0.08 | 51±13 | 46.7±0.4* |
| A29T | 129±26 | 0.89±0.11 | 115±28 | 51.9±0.5* |
| K32N | 134±7 | 0.52±0.17 | 76±18 | 51.6±0.3 |
| G34V | 6±1 | 0.32±0.11 | ~ 2 | N.Det. |
| E36K | 94±11 | 0.87±0.09 | 82±14 | 51.8±0.6 |
| S40L | 2±1 | 0.04±0.03 | > 1 | N.Det. |
| D41G | 7±2 | 0.18±0.23 | ~ 1 | 43.9±0.2 |
| D41Y | 2±1 | 0.21±0.14 | ~ 1 | 42.1±0.3 |
| M45L | 52±41 | 0.63±0.07 | 33±42 | 48.1±0.2 |
| M45I | 46±29 | 0.39±0.26 | 18±39 | 48.4±0.3 |
| I51V | 64±21 | 0.62±0.13 | 40±25 | 46.8±0.2 |
| W106R | 24±16 | 0.21±0.07 | ~ 5 | 45.4±0.5 |
| W106C | 66±45 | 0.32±0.08 | 21±45 | 49.3±0.5 |
| F107C | 95±64 | 0.14±0.10 | 13±65 | 52.4±0.5 |
| M155I | 35±33 | 0.14±0.11 | ~ 5 | 50.9±0.3 |
| H162N | 65±71 | 0.45±0.38 | 29±80 | 51.1±0.9 |

* From Pacheco-García JL, Cano-Muñoz M, Sánchez-Ramos I, Salido E, Pey AL. (2020). Naturally-Occurring Rare Mutations Cause Mild to Catastrophic Effects in the Multifunctional and Cancer-Associated NQO1 Protein. J. Pers. Med. 10, 207. doi: 10.3390/jpm10040207.
