# Supplementary material for: Effect of naturally-occurring mutations on the stability and function of cancer-associated NQO1: Comparison of experiments and computation
Source: Front Mol Biosci. 2022 Nov 24;9:1063620. doi: 10.3389/fmolb.2022.1063620 (PMC9730889; doi:10.3389/fmolb.2022.1063620)
Supplement: Supplementary file 3 [file Table2.DOCX]

**Supplementary Table 2. Variant effects on FAD binding.** The apparent dissociation constants (*K*_d FAD_) were determined by titration of apo-NQO1 variants with FAD. Data are best-fit parameters from at least two independent titrations. The local stability of the TCS (close to the FAD binding site) was determined by proteolysis with thermolysin (*k*_prot_). *k*_prot_ is the second-order rate constant for proteolysis obtained from the linear dependence of the apparent first-order rate constant on protease concentration.

| **Variant** | ***K*_d_ _FAD_ (nM)** | ***k*_prot_** **(µM·min^-1^)** |
| --- | --- | --- |
| WT | 16.1±2.7 | 0.149±0.024 |
| G3S | 23.2±5.4 | 0.146±0.013 |
| G3D | 13.9±5.2 | 0.111±0.032 |
| L7P | N.Det. | N.Det. |
| L7R | N.Det. | N.Det. |
| V9I | 23.3±6.5 | 0.101±0.009 |
| T16M | 174±31 | 1.075±0.115 |
| Y20N | 46.8±20.0 | 0.292±0.017 |
| A29T | 80.8±25.7 | 0.153±0.005 |
| K32N | 12.7±4.3 | 0.126±0.013 |
| G34V | N.Det. | N.Det. |
| E36K | 13.1±5.7 | 0.109±0.011 |
| S40L | N.Det. | N.Det. |
| D41G | N.Det. | 0.271±0.029 |
| D41Y | N.Det. | 0.754±0.072 |
| M45L | 8.4±8.2 | 0.054±0.006 |
| M45I | 9.3±6.9 | 0.086±0.025 |
| I51V | 143±32 | 1.521±0.032 |
| W106R | 7400±4000* | 0.023±0.002 |
| W106C | 15±5* | 0.864±0.285 |
| F107C | 2.7±2.2* | 0.046±0.008 |
| M155I | 722±173* | 10.75±1.26 |
| H162N | 441±74* | 0.138±0.018 |

* Unpublished work.
