# Supplementary material for: Effect of naturally-occurring mutations on the stability and function of cancer-associated NQO1: Comparison of experiments and computation
Source: Front Mol Biosci. 2022 Nov 24;9:1063620. doi: 10.3389/fmolb.2022.1063620 (PMC9730889; doi:10.3389/fmolb.2022.1063620)
Supplement: Supplementary file 4 [file Table3.DOCX]

**Supplementary Table 3. Allelic frequency of NQO1 mutations found in the gnomAD database and experimentally analysed in this work.** Frequencies are reported from the gnomAD v.2.1. using all or control samples. For sake of comparison, data on the common P187S polymorphism is also added.

| **Variation** | **Allelic frequency** | |
| --- | --- | --- |
|  | **All (141.456) samples** | **Control (60.146) samples** |
| **G3S** | 1.21·10^-5^ | 1.86·10^-5^ |
| **L7R** | 7.80·10^-5^ | 5.49·10^-5^ |
| **V9I** | 3.90·10^-5^ | 4.16·10^-5^ |
| **T16M** | 2.83·10^-5^ | 4.64·10^-4^ |
| **Y20N** | 2.12·10^-5^ | 1.83·10^-5^ |
| **K32N** | 7.96·10^-5^ | 4.57·10^-5^ |
| **G34V** | 3.98·10^-6^ | 9.14·10^-6^ |
| **E36K** | 6.37·10^-5^ | 3.33·10^-5^ |
| **S40L** | 1.99·10^-5^ | 9.14·10^-6^ |
| **D41G** | 3.98·10^-6^ | 9.14·10^-6^ |
| **I51V** | 6.37·10^-5^ | 3.33·10^-5^ |
| **W106R** | 2.79·10^-5^ | 4.57·10^-5^ |
| **F107C** | 1.77·10^-5^ | 0 |
| **P187S** | 2.47·10^-1^ | 2.55·10^-1^ |
